# Comparative efficacy of intralesional therapies for keloid scars: a network meta-analysis

**DOI:** 10.1080/07853890.2026.2619295

**Published:** 2026-03-20

**Authors:** Camila Sanchez Cruz, Angelo Magallanes Bajana, William Matheus de Araujo, Christopher Romero Ríos, Malaz El Zubair Mohamed Khalil, Jose Medina, Karla Perozo, Alexa Bentley, Ernesto Calderon Martinez, Candela Romano

**Affiliations:** a Universidad Nacional Autónoma de México, Ciudad de México, Mexico; b Universidad de Guayaquil, Guayaquil, Ecuador; c V.N. Karazin Kharkiv National University, Kharkiv, Ukraine; d Universidad de Defensa de Nicaragua 4 de mayo, Managua, Nicaragua; e Primary Health Care Corporation (PHCC), Doha, Qatar; f University of Zulia, Maracaibo, Venezuela; g Universidad Xochicalco-Campus Tijuana, Tijuana, Mexico; h University of Texas Health Science Center at Houston, Houston, TX, USA; i University of California, Irvine, Irvine, CA, USA

**Keywords:** 5-Fluorouracil, botulinum toxin, corticoids, keloid reduction, scar management, meta-analysis, review, intralesional therapies

## Abstract

**Background:**

Keloids are pathological scars causing pain, pruritus, and emotional distress. While common treatments exist, emerging options such as insulin and botulinum toxin A (BTX-A) are underrepresented in comparative analyses. This network meta-analysis (NMA) aimed to evaluate the efficacy and safety profiles of various intralesional therapies for keloid scars.

**Methods:**

A PRISMA-guided search across seven databases was conducted on July 2025 and preregistered on PROSPERO (CRD420251088758). Included studies were randomized, non-randomized, and crossover trials of intralesional therapies. Pairwise and NMA were conducted for efficacy (keloid size/volume reduction), recurrence, and safety (adverse effects). Treatment rankings were based on surface under the cumulative ranking curve (SUCRA) scores. Statistical significance was set at (*p* < 0.05).

**Results:**

From 51 studies (3234 participants, 23 interventions), 5-fluorouracil (5-FU) + corticoids significantly improved keloid reduction compared to corticoids alone (RR = 1.59; 95% CI: 1.31–1.92; *p* < 0.01), with 5-FU + corticoids + YAG:Laser (RR = 2.73; 95% CI: 1.36–5.48; *p* < 0.01) showing an even greater effect. SUCRA values indicated that 5-FU + corticoids + YAG:Laser (0.95), vitamin D + platelet rich plasma (0.83), and 5-FU + corticoids (0.78) had the highest probability of effectiveness. For recurrence, no significant differences were found across interventions, suggesting insufficient evidence. Regarding adverse effects, a significant risk reduction was found for BTX-A (RR = 0.29; 95% CI: 0.09–0.95; *p* = 0.04) and Verapamil (RR = 0.35; 95% CI: 0.14–0.84; *p* = 0.01) compared to corticoids; others showed no significant effects.

**Conclusion:**

This NMA provides a comparative overview, indicating that combination therapies, most notably 5-FU + corticoids + YAG:Laser, offer the greatest benefit. While data for novel agents (insulin and BTX-A) suggest potential, further research with standardized methodologies and longer-term follow-up is crucial to define optimal treatment algorithms.

## Introduction

A keloid is defined as a type of raised scar; these develop because of atypical healing processes after skin injury, in which scarring extends beyond the boundaries of the initial wound [1,2]. It has been shown that keloidal fibroblasts have increased proliferation and decreased rates of apoptosis, leading to an overproduction of collagen and cytokines [3,4]. Keloids can be both uncomfortable and pruritic, often leading to pain and tenderness. Additionally, the emotional toll that the patients can experience from the development of these conditions can affect their overall quality of life [5]. Researchers suggest that various factors, including genetic predisposition, skin injuries, and infections, among others, play a role in their development. Despite the variety of treatment options available, none have been established as the definitive standard of care. Various approaches, from non-invasive methods to surgical interventions, have been attempted in clinical settings [6]. The most recent evidence on intralesional therapies for keloids was restricted to comparing five interventions, limiting the information and conclusions [7]. Moreover, recently, insulin and botulinum toxin A (BTX-A) have shown potential in the management of keloids, with insulin proving to be more effective than BTX-A [8]. In our study, we aim to incorporate additional emerging therapies, involve a larger patient population, and broaden our data search to reach a stronger conclusion on the preferred treatment for keloid scars.

## Methods

This systematic review was conducted in accordance with the recommendations and criteria established by the Preferred Reporting Items for Systematic Reviews and Meta-Analyses (PRISMA) reporting guidelines [9]. The protocol was pre-registered at the International Prospective Register of Systematic Review (PROSPERO) with the identifier code CRD420251088758.

### Searching method

A comprehensive literature search was first performed on 3 July 2025, across PubMed MEDLINE, Cochrane, Scopus, Web of Science, EMBASE, CINAHL, and Google Scholar. Search terms included: ‘keloid’, ‘keloid scar’, ‘intralesional’, ‘injection’, and ‘botulinum’ (Supplementary Tables 1–7).

**Table 1.** General outcomes.

Author year	Country	Study design	Risk of bias	Sample size (*N*), mean age (years ± *SD*)	Intervention	Treatment number/duration (M)	Outcome
Saha A, et al. 2012 [22]	India	RCT	Some concerns	N:20 (34.7 ± 11) N:24 (32.96 ± 9.6)	5-FU; Corticoids	5/NA 4/NA	5-FU and triamcinolone showed similar efficacy in reducing keloid volume. Higher incidence of pain, ulceration, and pigmentation changes was observed with 5-FU.
Nagarur K, et al. 2016 [23]	India	RCT	Some concerns	N:33 (NA) N:33 (NA)	5-FU + Corticoids; Corticoids	8/NA 8/NA	The combination of 5-FU and triamcinolone produced a statistically superior response.
Monteiro R, et al. 2022 [24]	India	RCT	Some concerns	N:15 (NA) N:15 (NA)	5-FU; 5-FU + Corticoids	4/NA 4/NA	5-FU + TAC is a better regimen for patients sensitive to pain or other local reactions
Aggarwal A., et al. 2018 [25]	India	RCT	Some concerns	N:16 (NA) N:16 (NA) N:16 (NA) N:17 (NA) N:15 (NA)	Corticoids; Corticoids + Hyaluronidase; Corticoids + Radiofrequency; Radiofrequency; Verapamil	8/NA 8/NA 4/NA 4/NA 8/NA	TAC + hyaluronidase, TAC alone, and RF + TAC were all effective. TAC + hyaluronidase was better tolerated. Verapamil and RF alone were ineffective.
Belie O., et al. 2021 [26]	Nigeria	RCT	Some concerns	N:40 (30.09 ± 0.90) N:37 (30.09 ± 0.90)	Corticoids; Verapamil	6/NA 6/NA	Verapamil alone was less effective; it could be considered for small lesions or patients who cannot tolerate steroids.
YosipovitchG., et al. 2001 [27]	Singapore	NRCT	Serious concerns	N:10 (25.9 ± 9.9) N:10 (25.9 ± 9.9)	Corticoids; Corticoids + Cryotherapy	3/NA 3/NA	The combination of cryotherapy and triamcinolone suggests synergistic benefit.
Veena V., et al. 2024 [28]	India	RCT	Low	N:30 (41 ± 3.8) N:30 (44 ± 4.6)	5-FU + Corticoids; Corticoids	7/NA 7/NA	The 5-FU + TAC combination was superior and had slightly fewer adverse events.
Prabhu A., et al. 2012 [29]	India	RCT	Some concerns	N:14 (NA) N:15 (NA)	5-FU; Corticoids	4/NA 4/NA	5-FU showed slightly better efficacy with higher side effects. For resistant keloids, 5-FU might offer an edge.
Krishna S., et al. 2025 [30]	India	RCT	Some concerns	N:30 (32.60 ± 3.43) N:30 (34.21 ± 3.10)	Corticoids + Bleomycin; Corticoids + Cryotherapy	4/NA 4/NA	Bleomycin + TAC provided the most significant improvement with fewer side effects. Cryo + TAC was good but less safe.
Albalat W., et al. 2022 [31]	Egypt	RCT	Low	N:40 (33 ± 11.27) N:40 (33 ± 10.25) N:40 (31 ± 10.78) N:40 (32 ± 12.77)	5-FU; Corticoids; Platelet-rich plasma; Verapamil	6/NA 6/NA 6/NA 6/NA	Verapamil and PRP showed better overall scar improvement than the traditionally favored triamcinolone, while 5-FU was least effective.
Shahmoradi Z., et al. 2020 [32]	Iran	RCT	Some concerns	N:19 (38.00 ± 15.38) N:19 (32.63 ± 12.34)	Corticoids; Corticoids + Bevacizumab	3/NA 5/NA	Bevacizumab showed added benefit in height reduction. Triamcinolone alone had better pigmentation and total score improvements. Both are viable with slight differences.
Neinaa Y., et al. 2020 [33]	Egypt	RCT	Low	N:20 (25.4 ± 4.1) N:20 (26.1 ± 4.2) N:20 (23.5 ± 3.3)	BTX-A; Corticoids; Platelet-rich plasma	3/NA 3/NA 3/NA	BTX-A and PRP are superior to Triamcinolone in efficacy and safety.
Ismail S., et al. 2020 [34]	Egypt	RCT	High	N:35 (30.24 ± 10.72) N:34 (31.2 ± 12.61)	5-FU; BTX-A	6/NA 6/NA	BTX-A is statistically superior to 5-FU in both efficacy and tolerability, including recurrence control.
Cohen A., et al. 2023 [35]	USA	NRCT	Serious concerns	N:75 (NA) N:34 (NA)	Corticoids; Corticoids + Cryotherapy	NA	TAC + cryotherapy was more effective in reducing keloid size; pain and pruritus improvements were similar to TAC alone.
Chen X., et al. 2017 [36]	China	RCT	Some concerns	N:23 (26.5 ± 7.5) N:23 (26.5 ± 9.5) N:23 (27.2 ± 6.4)	5-FU + Corticoids; 5-FU + Corticoids + YAG laser; Corticoids	3/NA 3/NA 3/NA	Corticoid + 5-FU + Nd:YAG was the most effective, with significantly better symptom relief and higher ‘excellent’ response rates.
Hewedy E., et al. 2020 [37]	Egypt	RCT	Low	N:20 (23.71 ± 8.41) N:20 (29.05 ± 12.32)	Corticoids; Corticoids + Platelet-rich plasma	4/NA 4/NA	PRP + TAC was more effective in scar improvement and had fewer side effects than TAC alone.
Kaushal V., et al. 2020 [38]	India	RCT	Low	N:30 (NA) N:30 (NA)	Corticoids; Corticoids + Radiofrequency	6/NA 6/NA	Both treatments were similarly effective overall; TAC + RF had lower recurrence and greater score reduction.
Kumar P., et al. 2023 [39]	India	RCT	Some concerns	N:30 (NA) N:30 (NA)	5-FU + TAC; TAC + Verapamil	8/6 8/6	TAC + 5-FU was more effective and faster in reducing keloid parameters than TAC + verapamil, with no major safety differences reported.
Hoq A., et al. 2023 [40]	Bangladesh	NRCT	Serious concerns	N:15 (29.16 ± 8.79) N:15 (30.22 ± 9.15)	TAC; 5-FU + TAC	8/7 8/7	TAC + 5-FU is more effective and faster in response, with fewer side effects compared to TAC alone
Haghani-Dogahe Z., et al. 2023 [41]	Iran	RCT	High	N:21 (36.69 ± 8.95) N:21 (35.5 ± 8.27)	TAC; TAC + verapamil	8/4.5 8/4.1	TAC + verapamil More effective in treatment of keloid
Saki N., et al. 2019 [42]	Iran	RCT	Some concerns	N:15 (31.53 ± 12.58) N:15 (31.53 ± 12.58)	TAC; Verapamil	NA	Better improvement in height and pliability was seen with TAC than verapamil
Listiawan M., et al. 2019 [43]	Indonesia	RCT	Some concerns	NA	TAC; TAC + FRCO_2_	2/48 3/48	Both groups had significant collagen density reduction, but no statistically significant difference between them
Ali H., et al. 2020 [44]	Pakistan	RCT	Some concerns	N:30 (34.97 ± 8.05) N:30 (36.97 ± 8.53)	5-FU; 5-FU + TAC	11/12 11/12	Efficacy is better in combination group
Ali N., et al. 2021 [45]	Pakistan	RCT	Some concerns	N:75 (25.78 ± 8.54) N:75 (26.09 ± 8.21)	5-FU + TAC; TAC	4/12 4/12	The efficacy of intralesional 5-FU + TAC is significantly higher than TAC alone for the treatment of keloids.
Manzoor H., et al. 2020 [46]	Pakistan	RCT	Some concerns	N:30 (27.19 ± 9.76) N:30 (29.10 ± 9.47) N:30 (28.17 ± 8.69)	5-FU; TAC; 5-FU + TAC	6/6 6/6 6/6	The combination of 5-FU + TAC has better efficacy as compared to 5-FU alone group and TAC alone group in the management of keloids.
Khan S., et al. 2022 [47]	Multan	RCT	Some concerns	N:80 (31.77 ± 9.14) N:80 (29.77 ± 6.66)	TAC; 5-FU + TAC	8/3 8/3	The efficacy of intralesional 5-FU + TAC is significantly higher than TAC alone for the treatment of keloids
Qadir M., et al. 2025 [48]	Pakistan	RCT	Some concerns	N:25 (39.7 ± 14.1) N:25 (35.7 ± 9.2)	TAC; 5-FU	4/3 4/3	Intralesional 5-FU is significantly more effective than triamcinolone acetonide in reducing keloid height
Naseem S., et al. 2022 [49]	Pakistan	RCT	Some concerns	N:30 (33.6 ± 8.8) N:30 (32.1 ± 8.8)	TAC + 5-FU TAC	6/6 6/6	The combination of 5-FU with TAC is preferred over TAC alone for the treatment of keloids
Fayed S., et al. 2022 [50]	Egypt	RCT	Some concerns	N:20 (NA) N:20 (NA)	FRCO_2_ + Verapamil; FRCO_2_ + TAC	4/4 4/4	Combined fractional CO_2_ laser with intralesional triamcinolone therapy showed better clinical improvement compared to the combined fractional CO_2_ laser with intralesional verapamil therapy, but with more adverse effects
Srivastava S., et al. 2019 [51]	India	RCT	Some concerns	N:20 (32.65 ± 9.74) N:20 (30.45 ± 9.33) N:20 (29.45 ± 8.24)	FRCO_2_; Verapamil	8/6 8/6	All groups are efficient, but TAC has the fastest response in treating keloids when compared to other modalities.
Rana P., et al. 2024 [52]	India	RCT	Some concerns	N:30 (NA) N:30 (NA)	5-FU; 5-FU + TAC	NA	Combining 5-FU + TAC has a better response rate than 5-FU alone
Srivastava S., et al. 2018 [53]	India	RCT	Some concerns	N:20 (26.35 ± 6.11) N:20 (27.55 ± 8.54) N:20 (29.9 ± 10.19)	TAC; 5-FU; TAC + 5-FU	8/6 8/6 8/6	A combination of TAC + 5-FU seems to offer the balanced benefit of faster and more efficacious response with lesser adverse effects when compared to individual drugs.
Gamil H., et al. 2019 [54]	Egypt	RCT	Some concerns	N:26 (28.4 ± 6.5) N:26 (28.4 ± 6.5) N:24 (27.8 ± 6)	TAC; BTX-A; TAC + BTX-A	3/3 3/3 3/3	Combined intralesional steroid and BTX-A injection appears more effective and safer than either treatment alone, offering better outcomes with fewer side effects.
Pazyar N., et al. 2024 [55]	Iran	RCT	High	N:22 (35.23 ± 8.57) N:22 (35.23 ± 8.57)	Corticoids; Vitamin D	6/5 6/5	By examining the VSS scale in each of the groups, this scale decreased significantly in both groups after the intervention (*p* = 0.001), which was greater in the group receiving triamcinolone (*p* = 0.001).
Khan H., et al. 2019 [56]	Pakistan	RCT	Some concerns	N:82 (32 ± 12.77) N:82 (33 ± 10.12)	Bleomycin; Corticoids	6/6 6/6	Mean baseline POSAS score was 91 ± 10.98 *SD* check in Group A and 90 ± 10.85 *SD* in Group B. POSAS score after 24 weeks 26 *SD* ± 11.91 in Group and 34 ± 12.28 in Group
Sadeghina A., et al. 2012 [57]	Iran	RCT	High	N:20 (NA) N:20 (45 ± 8.2)	5-FU; Corticoids	3/3 3/3	Both groups showed improvement in all parameters, but the improvement was more significant in the 5-FU group (*p* < .05). No side effects were detected in either group.
Jannati P., et al. 2015 [58]	Iran	RCT	Some concerns	N: 20 NA N:20 NA N:20 NA N: 20 NA	Triamcinolone + Cryotherapy; Verapamil; Verapamil + Cryotherapy; Cryotherapy	NA	Intralesional triamcinolone acetonide with cryotherapy showed the most efficacies with more adverse effects, but intralesional verapamil with cryotherapy showed good efficacy with less adverse effects
Ahsan Q., et al. 2018 [59]	Bangladesh	RCT	Some concerns	N:20 NA N:30 NA	Corticoids; Corticoids + Cryotherapy	3/3 3/3	Each of the treatment was individually effective in the treatment of keloid
Galal S., et al. 2025 [60]	Egypt	RCT	Some concerns	N:15 (25.67 ± 6.37) N:15 (26.73 ± 6.25) N:15 (27 ± 7.28)	Corticoids; pentoxifylline; Vitamin D	5/3 5/3 5/3	highly a statistically significant differences between VSS before and after sessions regarding vascularity pliability height and pigmentation
Shaarawy E., et al. 2015 [61]	Egypt	RCT	Some concerns	N:12 (26.17 ± 9.70) N:12 (32.42 ± 13.24)	BTX-A; Corticoids	3/6 6/6	Botulinum toxin A: significant decrease in height of lesions, and in redness score compared with baseline with no significant difference in between both groups. Corticoids: significant decrease in the volume of the lesions after treatment in both groups Statistically significant improvement in softening in group A
Elwan N., et al. 2025 [62]	Egypt	RCT	Low	N:20 (29.85 ± 11.68) N:20 (22 ± 9.55) N:20 (24.55 ± 10.79)	Platelet rich plasma; Vitamin D Platelet rich plasma + Vitamin D	4/2 4/2 4/2	all groups demonstrated a significant reduction in VSS scores across the three treatment protocols compared to pre-treatment assessments
Elradi M., et al. 2025 [8]	Egypt	RCT	Some concerns	N: 21 NA N:21 NA N: 21 NA	BTX-A; Corticoids; Insulin	4/4 4/4 4/4	A statistically significant difference was observed in volume reduction among the groups, with insulin achieving a significantly greater reduction than BTX-A while steroids achieved a significantly greater reduction than both insulin and BTX-A
Serag-Eldin Y., et al. 2021 [63]	Egypt	RCT	Some concerns	N:10 (19 ± 13.8) N:10 (25.9 ± 11.2) N:10 (26.6 ± 11.7)	Corticoids; Corticoids + Pentoxifylline; Pentoxifylline	5/2.9 5/2.3 5/3.3	A significant improvement in VSS was detected in all groups. Significantly better improvements in keloid height, pliability, pain, and itching were detected in the TAC and combination groups than in the PTX group.
Rasaii S., et al. 2019 [64]	Iran	RCT	Some concerns	N:23 (23.3 ± 1.2) N:23 (23.3 ± 1.2)	Corticoids; Corticoids + BTX-A	3/3 3/3	There was no significant difference between treatment arms with respect to height, vascularization, pliability, and pigmentation scores. The addition of BTX-A resulted in significant symptomatic improvement of pain and pruritus as compared to intralesional triamcinolone alone (*p* < 0.001)
Mohamed B., et al. 2023 [65]	Egypt	RCT	Some concerns	N:40 (31.10 ± 9.13) N:40 (31.10 ± 9.13)	Corticoid; Enalapril	3/3 3/3	Both enalapril and TAC had the same clinical effect. Enalapril could be a safe alternative to steroids in the treatment of keloid and hypertrophic scars
Mittal S., et al. 2025 [66]	India	RCT	Some concerns	N:25 (31 ± 8) N:25 (30 ± 7) N:25 (28 ± 9) N:25 (33 ± 6)	5-FU + Corticoids; Corticoids; Corticoids + Topical silicone gel; Radiofrequency	4/2 4/2 4/2 4/2	Overall improvement maximum response was seen in group TAC + 5-FU and TAC + SGS was least effective.
Sharma S., et al. 2012 [67]	India	RCT	High	N:25 NA N:25 NA	5-FU; 5-FU + Corticoids	7/3 7/3 7/3	The combination of 5-FU and triamcinolone acetonide is a better modality of treatment of small keloids compared with 5-FU alone.
Ehsani A., et al. 2025 [68]	Iran	RCT	Some concerns	N:8 (37.63 ± 9.66) N:8 (37.75 ± 15.83)	5-FU + Corticoids + Hyaluronidase; Corticoids	3/3 3/3	triple therapy was as effective as steroid monotherapy, with better outcomes in specific aspects of scar improvement and without side effects. Hyaluronidase may be a promising area for further research
Danielsen P., et al. 2016 [69]	Australia	RCT	Some concerns	N:14 (32.1 ± 10.5) N:14 (32.1 ± 10.5)	Corticoids; Verapamil	4/4 4/4	Kaplan-Meier survival curve analysis demonstrated significantly higher recurrence rate in the verapamil-treated half of the scar at 12 months post-surgery
Saleem F., et al. 2017 [70]	Pakistan	RCT	Some concerns	N:50 (30.82 ± 8.20) N:50 (32.46 ± 6.85)	5-FU + Corticoids; Corticoids	3/3 3/3	FU + TAC was efficacious in 98% of cases (group A) and TAC alone in 62% of cases (group B). No serious adverse effects were noticed in either group.
Hietanen K., et al. 2018 [71]	Finland	RCT	Low	N:25 NA N:25 NA	5-FU; Corticoids	NA/6 NA/6	5-FU increased the proliferation rate and did not affect vascularity in the histopathology assessment. TAC, 5-FU no significant difference in treatment effectiveness

Abbreviations: RCT, randomized controlled trial; 5-FU, 5-fluorouracil; TAC, triamcinolone acetonide; BTX-A, botulinum toxin type A; PRP, platelet-rich plasma; PTX, pentoxifylline; Cryo, cryotherapy; RF, radiofrequency; FRCO_2_, fractional carbon dioxide laser; Nd:YAG, neodymium-doped yttrium aluminum garnet laser; VSS, Vancouver Scar Scale; POSAS, Patient and Observer Scar Assessment Scale; SGS, silicone gel sheet.

**Table 2.** Surface under the cumulative ranking curve (SUCRA) scores for intradermal interventions across effectiveness, recurrence, and adverse events.

Intervention	Effectiveness	Recurrence	Adverse events
5-FU	0.3587	0.3684	0.5946
5-FU + Corticoids	0.7798	0.5404	0.3838
5-FU + Corticoids + YAG:Laser	0.9511	—	—
Bleomycin	0.4836	—	—
Botulinum toxin type A	0.6187	0.6863	0.7815
Corticoids	0.2909	0.4410	0.2724
Corticoids + Bleomycin	—	0.7287	—
Corticoids + Botulinum toxin type A	—	0.5210	0.9193
Corticoids + Cryotherapy	0.6159	0.6788	—
Corticoids + Hyaluronidase	0.4466	—	0.4458
Corticoids + Pentoxifylline	0.3381	0.2446	0.3940
Corticoids + Radiofrequency	0.4690	0.6350	0.3477
Corticoids + Topical silicone gel	0.2214	0.2494	0.1217
Corticoids + Verapamil	—	—	0.6126
Enalapril	—	—	0.6256
Insulin	—	0.5375	0.3921
Pentoxifylline	0.0242	0.4546	0.4645
Platelet rich plasma	0.5781	0.6207	0.7030
Radiofrequency	0.2058	0.3902	0.3748
Verapamil	0.4885	0.2417	0.7420
Verapamil + Cryotherapy	0.5429	—	—
Vitamin D	0.7597	0.5840	0.3246
Vitamin D + Platelet rich plasma	0.8270	0.5776	—

Denote the top-ranked intervention in each category. ‘–’ indicates that the intervention was not assessed for that outcome.

### Eligibility criteria

We included randomized controlled trials (RCTs), non-randomized studies, and crossover trials published in English or Spanish, from database inception to the present. Eligible participants were patients of any age with keloid scars (defined as scar tissue extending beyond the wound margin) who had received any intralesional therapy. Studies limited to hypertrophic scars were excluded [10]. Only trials comparing intralesional therapy with standard care, placebo, or other interventions were analyzed. We accepted studies reporting efficacy outcomes. The primary outcome was reduction in scar size or volume (≥50%), and secondary outcomes included improvements in symptoms such as pain and itching, as well as adverse effects [11].

### Selection of studies

All references were exported to Rayyan (Rayyan Systems Inc., Cambridge, MA, USA), and duplicates were removed [12]. Two authors independently completed the eligibility assessment, first by title and abstract and, subsequently, by full-text assessment. In disagreements between reviewers, a third reviewer was appointed to reach a consensus.

### Data extraction

Two reviewers independently collected data, and any disagreements were resolved by discussion; when needed, a third reviewer mediated to reach consensus. Standardized procedures were applied for data extraction, supported by specialized tools such as WebPlotDigitizer (Autometrics, Austin, TX, USA) for digitizing figures, the Cochrane Calculator for statistical conversions, and StatsToDo for complex calculations. Extracted variables included study characteristics (e.g. country of origin, type of intervention, dose, and treatment frequency), as well as risk of bias information [13–15].

### Assessment of risk of bias in included studies

The quality of included studies was evaluated according to the Cochrane recommendations. For randomized controlled trials (RCTs), the Cochrane RoB 2.0 tool was used [16], while for non-randomized studies, the ROBINS-I tool (Risk of Bias in Non-randomized Studies of Interventions) [16]. Two independent reviewers evaluated the risk of bias. Any reviewer discrepancies were resolved through discussion with a third blinded reviewer.

### Statistical analysis

All analyses were conducted in R version 3.4.3 [17]. Pooled estimates were calculated using a random-effects model based on the DerSimonian–Laird method. When data were insufficient for meta-analysis, findings were summarized qualitatively [18]. Effect measures were reported as relative risk (RR) or standardized mean difference (SMD) with 95% confidence intervals (CI). Statistical heterogeneity was assessed using the *I*^2^ statistic, with thresholds of <25, 25–50, and >50% interpreted as low, moderate, and high heterogeneity, respectively [19]. Network geometry and treatment rankings were explored through network plots, with ranking probabilities expressed as P-scores or SUCRA values. Forest plots were used to display comparisons across outcomes. Sensitivity analyses were performed to examine the influence of individual studies on pooled effects, incorporating risk-of-bias considerations [20].

## Results

### Study selection

In our initial search, we identified 2632 potential articles across seven databases. After removing 1165 duplicate articles, we conducted a screening of 1467 based on title and abstract, leading to the exclusion of 1335 articles. Fifty of the 132 articles sought for retrieval were not retrieved, leaving 82 articles for eligibility assessment. These remaining articles underwent screening. Ultimately, 51 studies were included in this review (Supplementary Figure 1) [21].

### Characteristics of included studies

The 51 included studies encompassed a total of 3234 participants. The majority of studies were conducted in India (27.5%), followed by Egypt (21.6%), Iran (17.6%), Pakistan (15.7%). Of these, 48 studies were randomized controlled trials (RCTs), while 3 used other study designs. All aimed to evaluate the efficacy and safety of intralesional therapies for keloid scar treatment. The interventions assessed across the included studies were diverse, with corticosteroids being the most frequently evaluated treatment, present in 49 studies. Combination therapies involving corticosteroids were common, including corticosteroids plus 5-Fluorouracil (5-FU) in 17 studies, corticosteroids plus cryotherapy in 5 studies, and corticosteroids combined with platelet-rich plasma (PRP) in 1 study. Other frequently studied interventions included 5-FU alone (12 studies), verapamil (10 studies), botulinum toxin type A (6 studies), and radiofrequency CO_2_ (FRCO_2_) in combination with corticosteroids (2 studies). Injection-related pain was the most common adverse effect reported across all treatment groups. Newer agents such as insulin and botulinum toxin A, although evaluated in a limited number of trials, showed promising efficacy with mild side effects. The information provided above is summarized in Table 1 [8,22–71].

### Risk of bias assessment

1. Among the 48 RCTs, 36 studies (75%) were categorized as having some concerns, while 7 studies (14,6%) were rated as low risk of bias. Notably, 5 studies (10,4%) were identified as having a high risk of bias (Supplementary Figure 2). All three non-RCT included studies (100%) were judged to have a serious risk of bias (Supplementary Figure 3).

### Meta analysis

#### Primary outcomes

**Figure 1.**
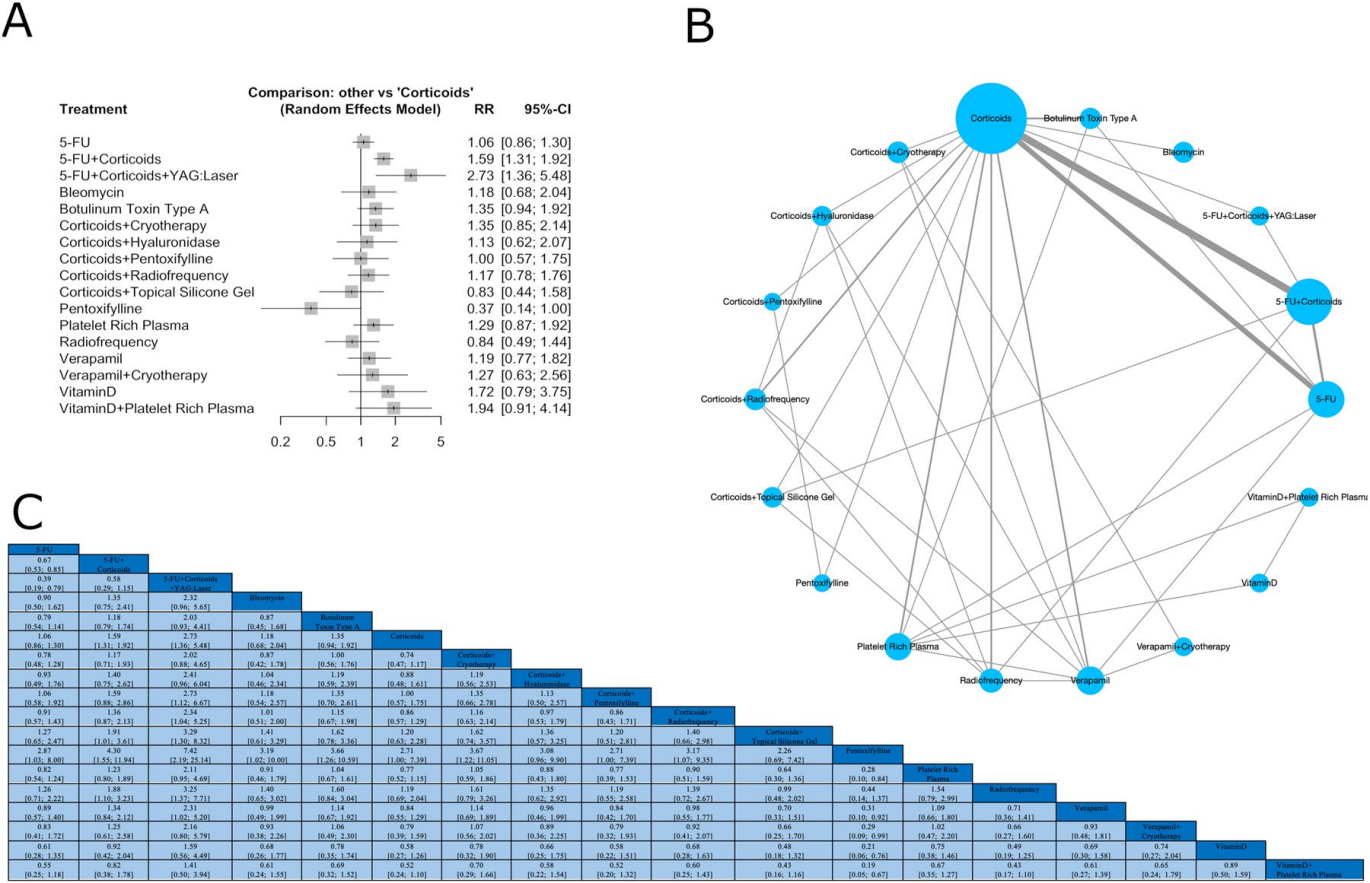
Network plot of treatment comparisons in the network meta-analysis for effectiveness. (A) The network plot illustrates treatment comparisons in the meta-analysis. Nodes (blue circles) represent interventions, with larger nodes indicating more participants. Edges (gray lines) denote direct comparisons, where thicker lines reflect a higher number of studies. The largest node represents the most frequently studied intervention, often the reference treatment. This visualization highlights the structure of available evidence, showing how treatments are connected and where direct comparisons exist. (B) Forest plot showing standardized relative risk (RR) with 95% confidence intervals for interventions *versus* corticoids in effectiveness. Positive RR favors the intervention. Squares represent effect sizes, and horizontal lines indicate confidence intervals. (C) League table with values for each intervention comparison.

**Figure 2.**
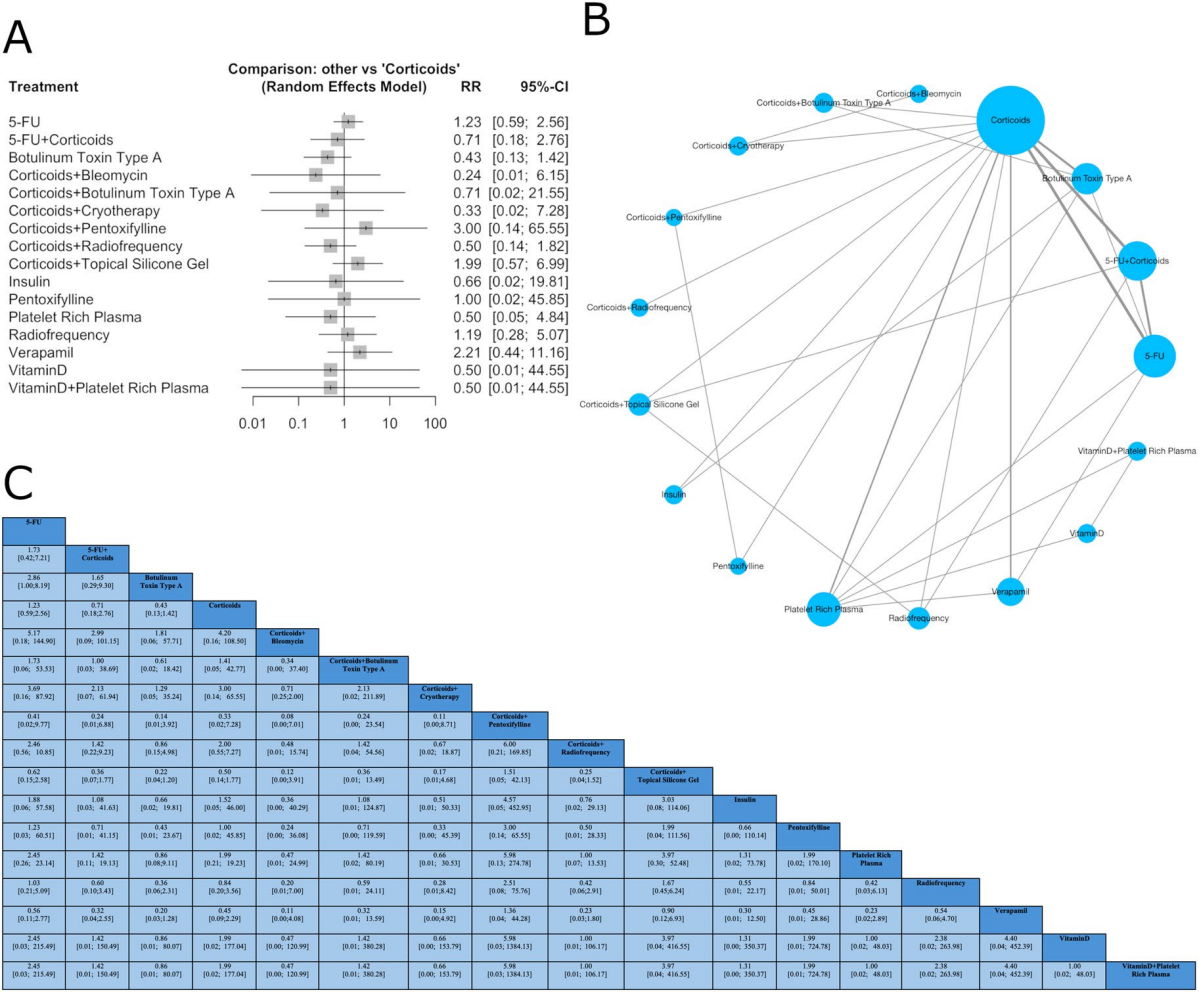
Network plot of treatment comparisons in the network meta-analysis for recurrence. (A) The network plot illustrates treatment comparisons in the meta-analysis. Nodes (blue circles) represent interventions, with larger nodes indicating more participants. Edges (gray lines) denote direct comparisons, where thicker lines reflect a higher number of studies. The largest node represents the most frequently studied intervention, often the reference treatment. This visualization highlights the structure of available evidence, showing how treatments are connected and where direct comparisons exist. (B) Forest plot showing standardized relative risk (RR) with 95% confidence intervals for interventions *versus* corticoids in recurrence. Negative RR favors the intervention. Squares represent effect sizes, and horizontal lines indicate confidence intervals. (C) League table with values for each intervention comparison.

**Figure 3.**
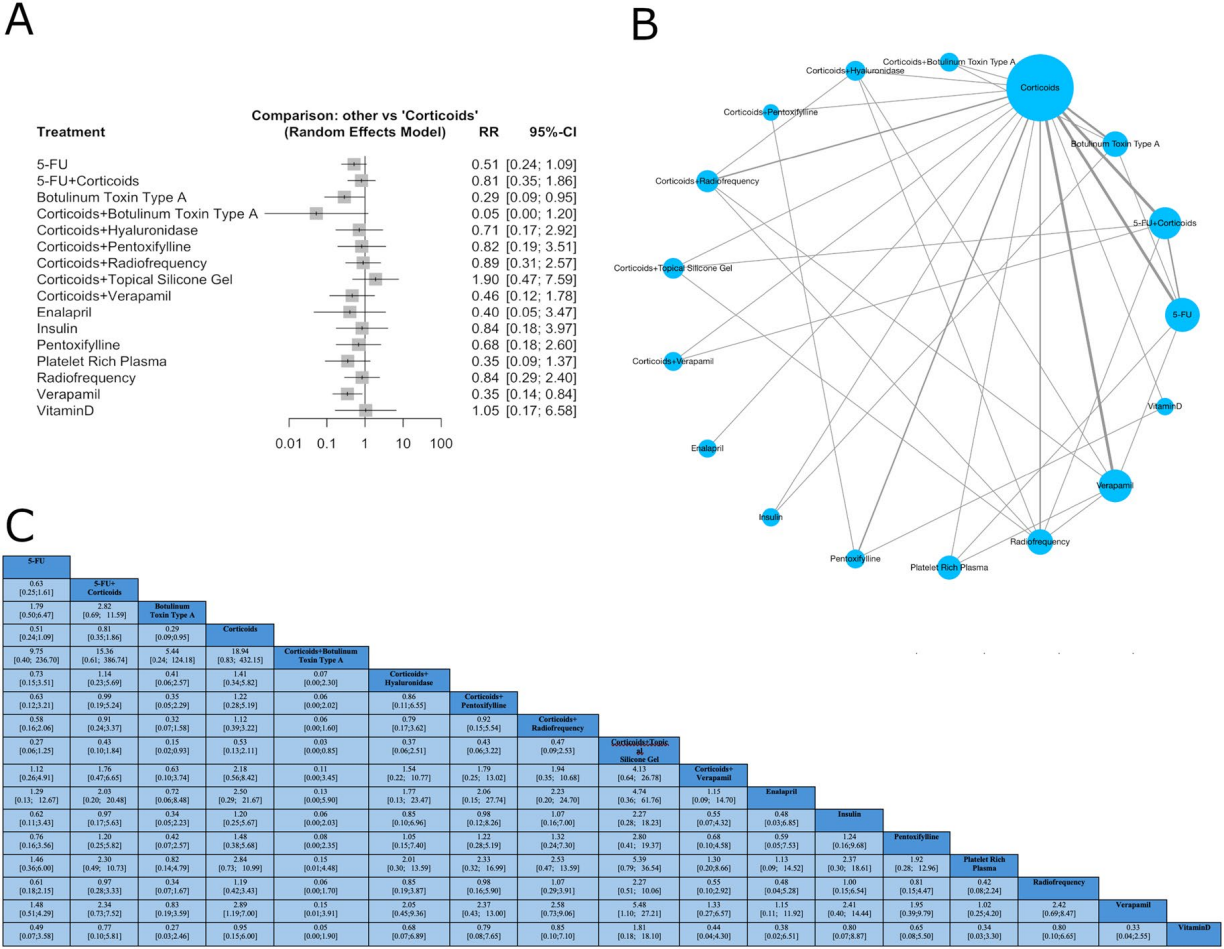
Network plot of treatment comparisons in the network meta-analysis for adverse events. (A) The network plot illustrates treatment comparisons in the meta-analysis. Nodes (blue circles) represent interventions, with larger nodes indicating more participants. Edges (gray lines) denote direct comparisons, where thicker lines reflect a higher number of studies. The largest node represents the most frequently studied intervention, often the reference treatment. This visualization highlights the structure of available evidence, showing how treatments are connected and where direct comparisons exist. (B) Forest plot showing standardized relative risk (RR) with 95% confidence intervals for interventions *versus* corticoids in recurrence. Negative RR favors the intervention. Squares represent effect sizes, and horizontal lines indicate confidence intervals. (C) League table with values for each intervention comparison.

The network meta-analysis (NMA) included 28 studies for effectiveness (Figure 1), 19 studies for recurrence (Figure 2), and 21 studies for adverse events (Figure 3), encompassing 18 interventions for effectiveness and 17 for recurrence and adverse events.

#### Effectiveness

The network meta-analysis revealed a statistically significant effect for the combination of 5-FU and corticoids compared to corticoids (RR = 1.59; 95% CI: 1.31–1.92; *p* < 0.0001), and for 5-FU + corticoids + YAG:Laser (RR = 2.73; 95% CI: 1.36–5.48; *p* = 0.0047; Figure 1). However, the overall analysis demonstrated substantial heterogeneity and inconsistency across the network (*I*^2^ = 68.9% [95% CI: 53.0%–79.4%]) within 5-FU *vs.* corticoids, and those involving radiofrequency, BTX-A, and hyaluronidase were particularly influential in driving heterogeneity and inconsistency. However, adjusting for differences in treatment comparisons eliminated inconsistency (*Q* = 19.82, *df* = 13, *p* = 0.0998). Despite overall network coherence, node-split analysis revealed significant local inconsistency in comparisons such as corticoids + radiofrequency *vs.* verapamil, and corticoids + hyaluronidase *vs.* verapamil between direct and indirect estimates, suggesting potential bias or effect modification in these treatment pathways (Supplementary Table 8, Supplementary Figures 4 and 5). Surface under the cumulative ranking curve (SUCRA) values indicated that 5-FU + corticoids + YAG:Laser (0.95), vitamin D + PRP (0.83), and 5-FU + corticoids (0.78) had the highest probability of being the most effective treatments. Rank probability plots further supported these findings (Table 2, Supplementary Figure 6). The league table of standardized mean differences (Figure 1) presents comparative effect estimates with 95% confidence intervals. The funnel plot (Supplementary Figure 7) visual inspection suggests asymmetry, with studies dispersed unevenly around the central effect estimate. Egger’s test confirmed significant small-study effects (*p* = 0.03), indicating possible publication bias.

#### Recurrence

The network meta-analysis did not reveal statistically significant differences for any intervention compared to corticoids (Figure 2), indicating no significant treatment effect. Importantly, the network demonstrated no evidence of heterogeneity or inconsistency. The estimated heterogeneity was null (*I*^2^ = 0% [95% CI: 0.0%–55.0%]). Neither within-design (*p* = 0.99) nor between-design (*p* = 0.88) inconsistency was observed. Node-split analysis did not reveal statistically significant local inconsistency across the network (all *p*-values > 0.05), supporting overall coherence (Supplementary Table 9, Supplementary Figures 7 and 8). SUCRA values indicated that corticoids + bleomycin (0.73), BTX-A (0.69), and corticoids + cryotherapy (0.68) had the highest probabilities of being the most effective treatments. Rank probability plots supported these findings, with standard treatments such as corticoids (0.44) and 5-FU (0.37) occupying intermediate to lower positions in the ranking distribution (Table 2, Supplementary Figure 9). The league table of standardized mean differences (Figure 2) presents comparative effect estimates with 95% confidence intervals. The funnel plot (Supplementary Figure 10) visual inspection suggests symmetry. Egger’s test confirmed no publication bias or small study effect (*p* = 0.10).

#### Adverse effects

The network meta-analysis revealed a statistically significant reduction in risk for BTX-A (RR = 0.29; 95% CI: 0.09–0.95; *p* = 0.0408) and verapamil (RR = 0.35; 95% CI: 0.14–0.84; *p* = 0.0192) compared to corticoids (Figure 3). However, substantial heterogeneity and inconsistency were detected across the network (*I*^2^ = 69.5% [95% CI: 50.5%–81.2%]). Design-specific decompositions identified corticoids *vs.* 5-FU + corticoids (*p* < 0.01) and corticoids *vs.* 5-FU (*p* = 0.03) as particularly influential. Multiple comparisons remained inconsistent after detachment of specific designs, such as corticoids *vs.* verapamil (*p* < 0.01) and 5-FU *vs.* BTX-A(*p* < 0.01), suggesting notable local inconsistency. Nonetheless, under the design-by-treatment interaction model, the network regained coherence (*p* = 0.98), indicating that accounting for interaction effects resolved global inconsistency. Node-split analysis revealed no statistically significant local inconsistency across the network (all *p*-values > 0.05), supporting the coherence between direct and indirect evidence (Supplementary Table 10, Supplementary Figures 11 and 12). SUCRA values for adverse events indicated that corticoids + BTX-A (0.92), BTX-A (0.78), and verapamil (0.74) had the highest probability of being the safest treatments. Rank probability plots supported these findings (Table 2, Supplementary Figure 13). The league table of standardized mean differences (Figure 3) presents comparative effect estimates with 95% confidence intervals. The funnel plot (Supplementary Figure 14) visual inspection suggests symmetry. Egger’s test confirmed no publication bias or small study effect (*p* = 0.06).

### Subgroup analysis

A subgroup analysis for recurrence was not necessarily due to low heterogeneity. A subgroup network meta-analysis of adverse event outcomes included 24 studies (*k* = 24), analyzed under a random-effects model (Supplementary Figure 15). Statistically significant increases in effectiveness were only observed for 5-FU + corticoids (RR = 1.59; 95% CI: 1.30–1.95; *p* < 0.0001) and 5-FU + corticoids + YAG:Laser (RR = 2.75; 95% CI: 1.36–5.57; *p* = 0.0051), compared to corticoids alone. Heterogeneity remained substantial (*I*^2^ = 69% [95% CI: 51.4%–80.2%]). According to SUCRA rankings, 5-FU + corticoids + YAG:Laser (0.95), vitamin D + PRP (0.81), and 5-FU + corticoids (0.78) had the highest probability of being associated with adverse events. The league table confirmed these trends, with consistent relative risks across direct and indirect comparisons. A subgroup network meta-analysis of adverse event outcomes included 24 studies (*k* = 24; Supplementary Figures 16 and 17), analyzed under a random-effects model. Only verapamil reached statistical significance (RR = 0.33; 95% CI: 0.13–0.86; *p* = 0.023), indicating a potential protective effect. Substantial heterogeneity persisted across the network (*I*^2^ = 73.8% [95% CI: 56.4%– 84.3%]). According to SUCRA rankings, corticoids + BTX-A (0.91), verapamil (0.74), and BTX-A (0.70) had the highest probability of being associated with the fewest adverse events.

The rank probability distributions and the league table were supported by consistent estimates across both direct and indirect comparisons (Supplementary Tables 11–14).

## Discussion

This network meta-analysis evaluated the comparative effectiveness and safety of 23 intralesional therapies for keloid scars. By including 51 randomized controlled trials and over 3000 participants, this review improves statistical power and addresses limitations of prior meta-analyses, such as a narrow treatment scope, absence of SUCRA-based rankings, and lack of adverse event reporting.

Although intralesional corticosteroids remain the most widely recommended intervention in clinical guidelines [72], they offer limited durability and may result in adverse effects such as atrophy and telangiectasia [73]. The combination of 5-FU, corticosteroids, and YAG:Laser significantly outperformed all other treatments in our analysis in efficacy rankings (SUCRA = 0.98, *p* < 0.01). On the other hand, medications such as BTX-A and verapamil had the lowest rates of adverse events. Notably, recurrence rates showed no statistically significant differences between treatments, potentially due to short follow-up durations and inconsistent definitions across studies. Nonetheless, the results reinforce the superiority of combination therapies for clinical response and highlight safer alternatives for patients at risk of side effects.

These findings extend the scope of prior meta-analyses. For example, Ren et al. [74] conducted a pairwise meta-analysis comparing triamcinolone (TAC) *vs.* TAC + 5-FU, pooling four RCTs (*n* = 256), and found significantly greater improvement in scar height (MD = −0.14, *p* = 0.002) and erythema (MD = −0.20, *p* = 0.004) in the combination group. In our NMA, TAC + 5-FU also ranked second in efficacy (SUCRA = 0.89) and mid-tier in safety (0.63), confirming its robust performance across outcomes. However, Ren et al. did not evaluate recurrence, and their analysis lacked comparison against other combination therapies, such as TAC + 5-FU + laser, which our analysis identified as the most effective [74]. Same as Yang et al. [7], whose network meta-analysis was limited to five intralesional therapies of keloids and concluded that combination treatments-particularly TAC plus 5-FU—demonstrate superior efficacy when compared to monotherapies. BTX-A showed comparable effectiveness to the TAC + 5-FU combination. Both our study and the study of Yang et al. point to the conclusion that combination therapies may offer more robust treatment responses. Regarding adverse events, Yang et al. analyzed seven studies involving BTX-A, TAC + 5-FU, TAC, and 5-FU, in which they found no significant differences among these treatments. Verapamil was not included in the safety analysis, limiting conclusions about its tolerability.

Bi et al. [75] and Li and Jin et al. [76] also align partially with our findings while highlighting important contrasts. Notably, Bi et al. examined BTX-A across 15 studies; however, most did not specify the type of scar tissue, and when specified, the scars were primarily hypertrophic. In contrast, our analysis focuses specifically on keloid scars, elucidating a distinct population. Nonetheless, their findings—reporting significant improvements in pain and scar thickness (OR = 2.51, *p* < 0.01)—remain consistent with our ranking of BTX-A as the second-safest intervention (SUCRA = 0.82), supporting the broader efficacy observed in the literature. However, we found BTX-A substantially less effective in scar reduction (SUCRA = 0.48), and without conclusive effects on recurrence, outcomes were not assessed in their analysis. Similarly, Li and Jin et al. studied verapamil and reported comparable response rates to steroids (RR = 0.97) but significantly fewer adverse events (RR = 0.42), aligning with our identification of verapamil as the safest agent (SUCRA = 0.88). However, our analysis placed verapamil among the least effective options (SUCRA = 0.32) and, consistent with their findings, showed no advantage in recurrence reduction (SUCRA = 0.57). These results suggest that while verapamil is well tolerated, it is better suited as an alternative for patients prioritizing safety rather than as a primary treatment choice.

Our findings support a more nuanced and evidence-driven approach to intralesional therapy selection for keloid scars. The consistent superiority of combination regimens suggests that monotherapy, although still widely used, may no longer be considered optimal as a first-line intervention. The identification of BTX-A and verapamil as the safest options provides valuable alternatives for patients with contraindications to steroids or high risk for adverse events. Importantly, the lack of meaningful differences in recurrence across interventions highlight the urgent need to integrate long-term follow-up and maintenance strategies into standard care.

### Limitations and practical implications

The lack of direct evidence for several treatment pairs resulted in the notable dependence on the transitivity assumption, which can put the evidence at risk for undetected biases and reduce the certainty of network estimates. Despite rigorous statistical modeling efforts that successfully resolved global inconsistencies across the network, the presence of considerable heterogeneity and local inconsistency was observed for efficacy and adverse events. This was particularly evident for specific efficacy comparisons (e.g. corticoids + radiofrequency *vs.* verapamil). This discordance between direct and indirect evidence suggests potential transitivity violations and that unmeasured confounders or variations in study characteristics could not be fully accounted for, reducing the confidence of specific comparative effects for these outcomes. Additionally, the initial substantial heterogeneity observed for adverse events could also reflect varied reporting standards, which warrants cautious interpretation of their safety profiles. Other limitations related to data availability and statistical power were also identified. This underscores the importance of conducting further high-quality clinical trials to validate current treatments and inform future clinical practice. Moreover, expanding research to include emerging therapeutic approaches may strengthen the evidence base needed to support more confident consideration of less established treatments.

## Conclusion

This analysis provides a comprehensive synthesis of current evidence on intralesional therapies for the treatment of keloid scars, identifying the combination of 5-fluorouracil, corticosteroids, and YAG:Laser as the most effective regimen in achieving effectiveness. Dual combinations outperformed monotherapies, reaffirming the clinical value of multimodal approaches. Although conventional agents remain foundational, emerging therapies like insulin and BTX-A have demonstrated encouraging results. However, the variability in study design, outcome reporting, and follow-up periods, alongside reliance on indirect comparisons, underscores the need for high-quality, adequately powered randomized trials.

## Supplementary Material

PRISMA Checklist.docx

Supplementary Materials.docx

## Data Availability

The data supporting the findings of this study are available within the referenced articles included in the analysis. Additional datasets generated and analyzed during the current study (e.g. extracted data and analysis code) are available from the corresponding author on reasonable request, in accordance with the Taylor & Francis Share Upon Reasonable Request policy (https://authorservices.taylorandfrancis.com/data-sharing-policies/share-upon-reasonable-request/).
