# Supplementary material for: Comparative efficacy of intralesional therapies for keloid scars: a network meta-analysis
Source: Ann Med. 2026 Mar 20;58(1):2619295. doi: 10.1080/07853890.2026.2619295 (PMC13007407; doi:10.1080/07853890.2026.2619295)
Supplement: Supplementary Materials.docx [file IANN_A_2619295_SM5626.docx]

**Figures**

**Supplementary Figure 1. PRISMA 2020 Flow Diagram of Study Selection**


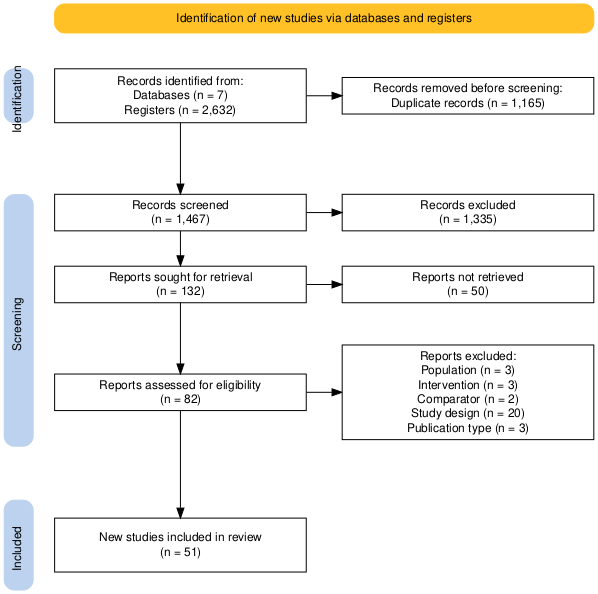


Flow diagram illustrating the study selection process for the systematic review. A total of 2,632 records were identified through searches of seven databases. After removing 1,165 duplicates, 1,467 records were screened by title and abstract. Of these, 132 full-text articles were sought, but 50 could not be retrieved. Among the 82 assessed for eligibility, 31 were excluded based on predefined criteria (population: n = 3; intervention: n = 3; comparator: n = 2; study design: n = 20; publication type: n = 3). In total, 51 studies were included in the systematic review. This process followed PRISMA 2020 guidelines.

**Risk of Bias within Studies**

**Supplementary Figure 2. Risk of bias assessment of randomized controlled trials using the Cochrane Risk of Bias 2.0 tool.**


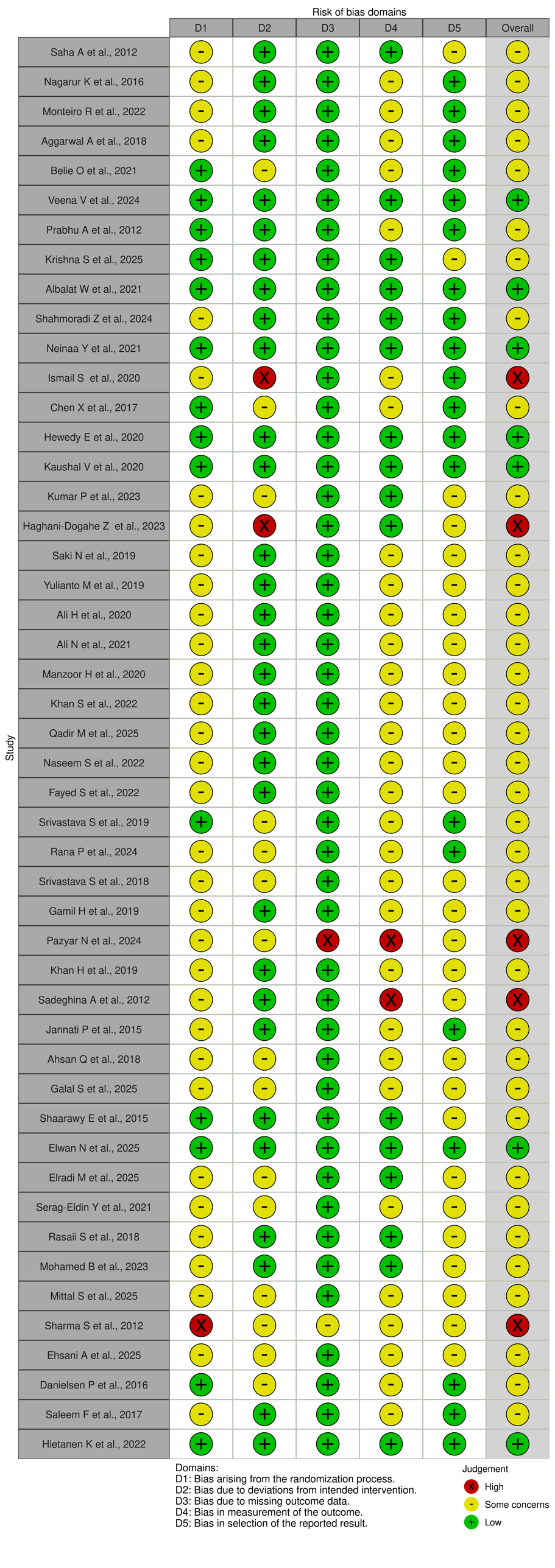


Five domains were evaluated: D1 (bias arising from the randomization process), D2 (bias due to deviations from intended interventions), D3 (bias due to missing outcome data), D4 (bias in measurement of the outcome), and D5 (bias in selection of the reported result). The overall risk of bias reflects the most severe judgment across domains. Judgments are color-coded as follows: green = low risk, yellow = some concerns, red = high risk.

**Supplementary Figure 3. Risk of bias assessment for non-randomized studies using ROBINS-I tool.**


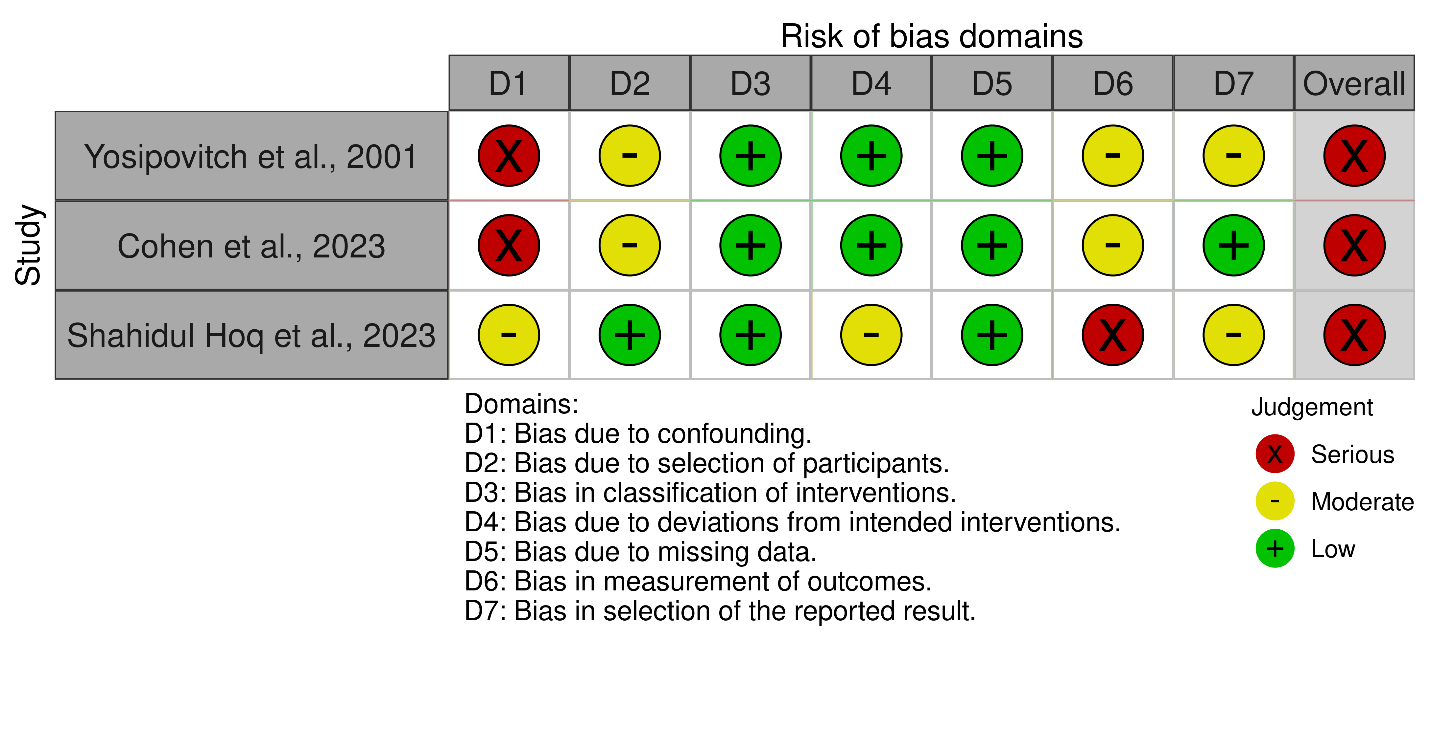


Risk of bias across seven ROBINS-I domains: D1 (confounding), D2 (participant selection), D3 (intervention classification), D4 (deviation from intended intervention), D5 (missing data), D6 (outcome measurement), D7 (reported result selection). Judgment was classified as Low (+), Moderate (–), or Serious (X). Overall risk of bias was based on the most severe judgment across domains.

**Supplementary Figure 4. Direct and Indirect Evidence Proportions in Network Meta-Analysis for Effectiveness.**
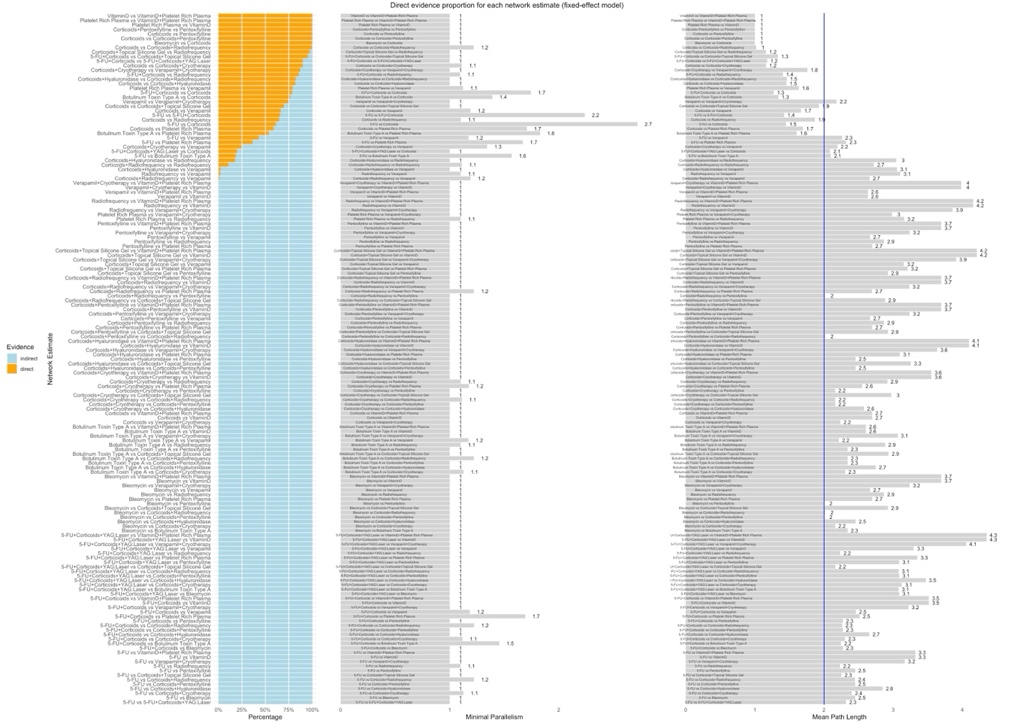


This figure presents the proportion of direct (orange) and indirect (blue) evidence for each network estimate, along with minimal parallelism and mean path length metrics in a fixed-effect model. The left panel shows the percentage of direct versus indirect contributions. The middle panel represents minimal parallelism values, indicating the minimum number of independent paths contributing to each comparison. The right panel displays the mean path length, reflecting the average number of steps connecting treatments in the network. A lower mean path length suggests stronger direct evidence.

**Supplementary Figure 5. Node-Split Analysis of Direct and Indirect Evidence for Effectiveness**


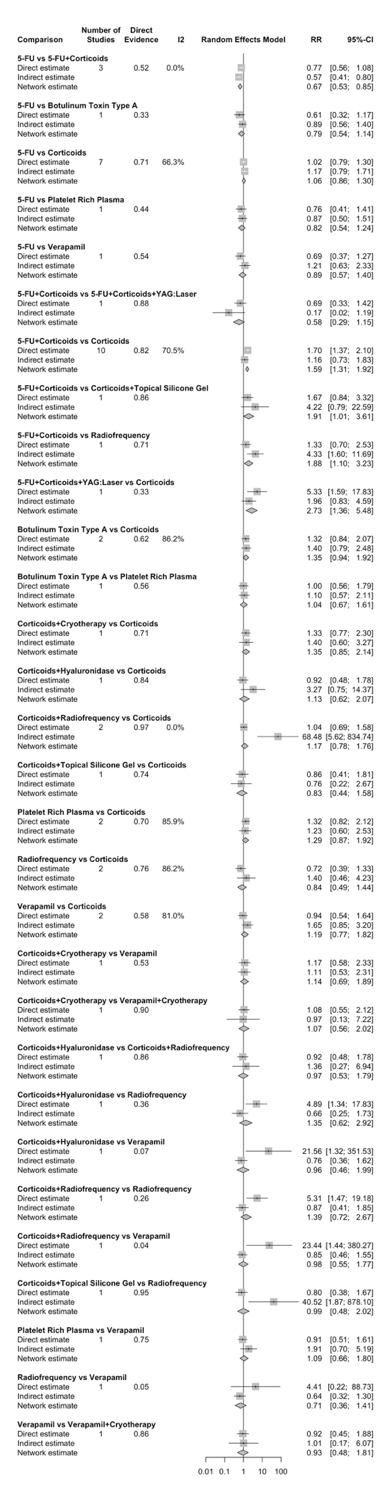


This node-split analysis compares direct, indirect, and network estimates of Relative Risk (RR) with 95% confidence intervals (CI) for effectiveness in the treatment of keloid scars. Direct estimates (squares) come from head-to-head studies, indirect estimates are inferred through network meta-analysis, and network estimates (diamonds) combine both. Differences between direct and indirect estimates indicate potential network inconsistency.

**Supplementary Figure 6. Rank Probability Distributions for Effectiveness**


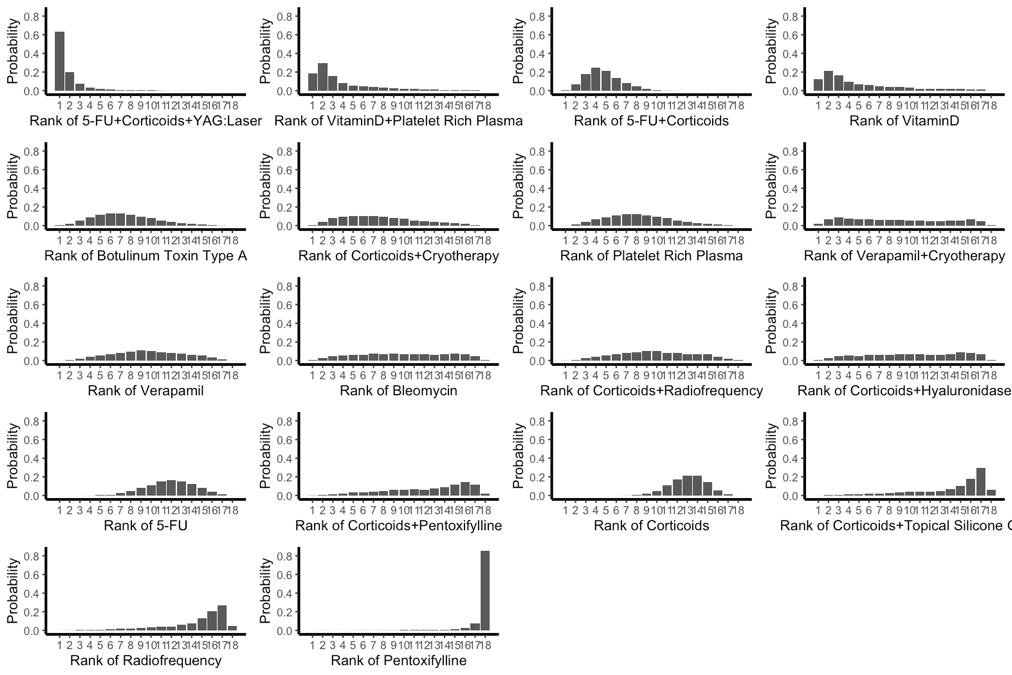


This figure presents the rank probability distributions of the included interventions for effectiveness based on network meta-analysis. Each panel shows the probability of an intervention achieving a particular rank, with lower ranks indicating higher effectiveness. A left-skewed distribution suggests a higher likelihood of being among the top-ranked treatments, while a right-skewed distribution indicates a higher probability of being among the least effective.

**Supplementary Figure 7. Funnel Plot for Publication Bias in Network Meta-Analysis of Effectiveness**


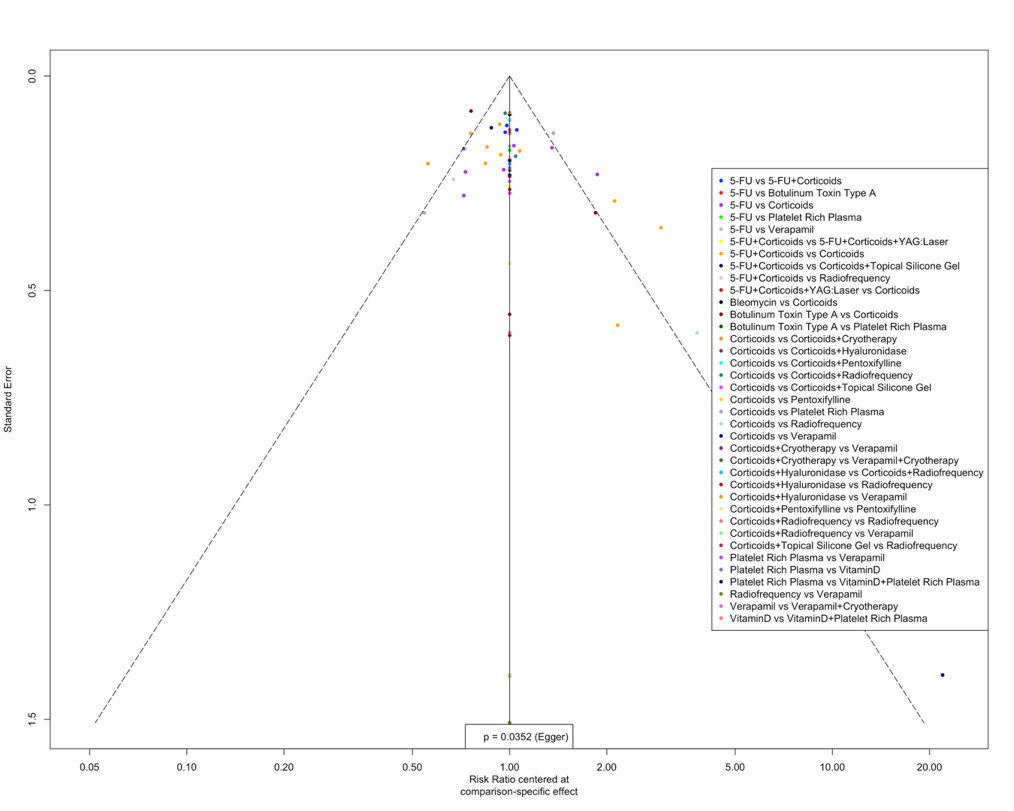


This funnel plot assesses small-study effects and potential publication bias in the network meta-analysis. The x-axis represents the Relative Risk centered at the comparison-specific effect, while the y-axis shows the standard error. Each point represents a treatment comparison, color-coded according to the legend. The dashed lines indicate the 95% confidence region. The presence of asymmetry suggests possible publication bias, supported by Egger’s test (p = 0.035).

**Supplementary Figure 8. Direct and Indirect Evidence Proportions in Network Meta-Analysis for Recurrence.**


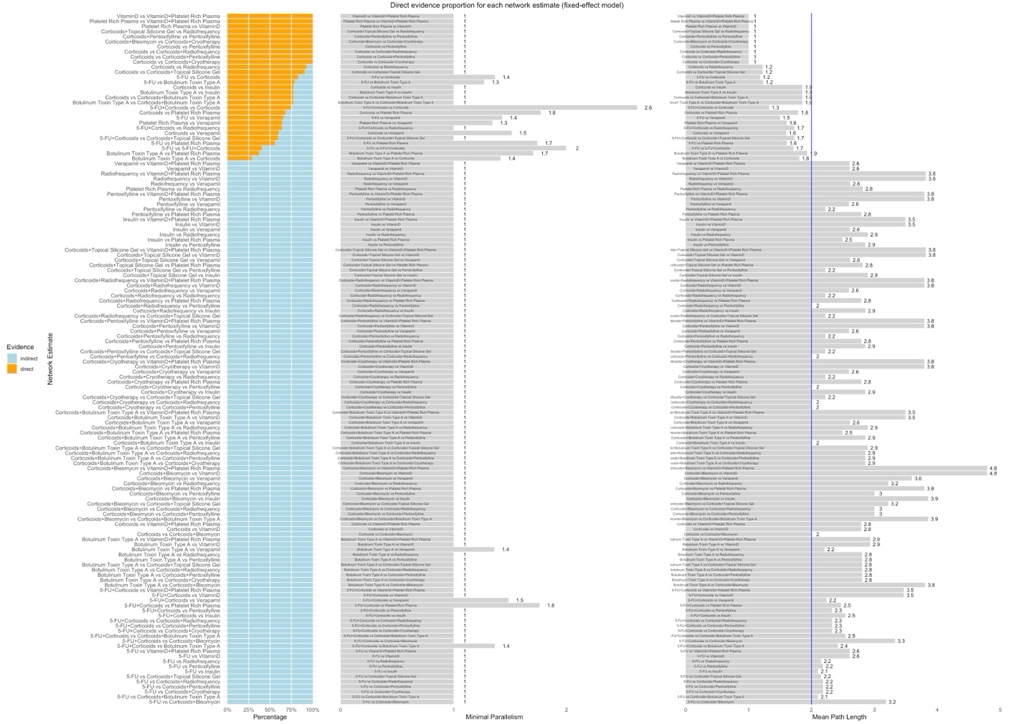


This figure presents the proportion of direct (orange) and indirect (blue) evidence for each network estimate, along with minimal parallelism and mean path length metrics in a fixed-effect model. The left panel shows the percentage of direct versus indirect contributions. The middle panel represents minimal parallelism values, indicating the minimum number of independent paths contributing to each comparison. The right panel displays the mean path length, reflecting the average number of steps connecting treatments in the network. A lower mean path length suggests stronger direct evidence.

**Supplementary Figure 9. Node-Split Analysis of Direct and Indirect Evidence for Recurrence**


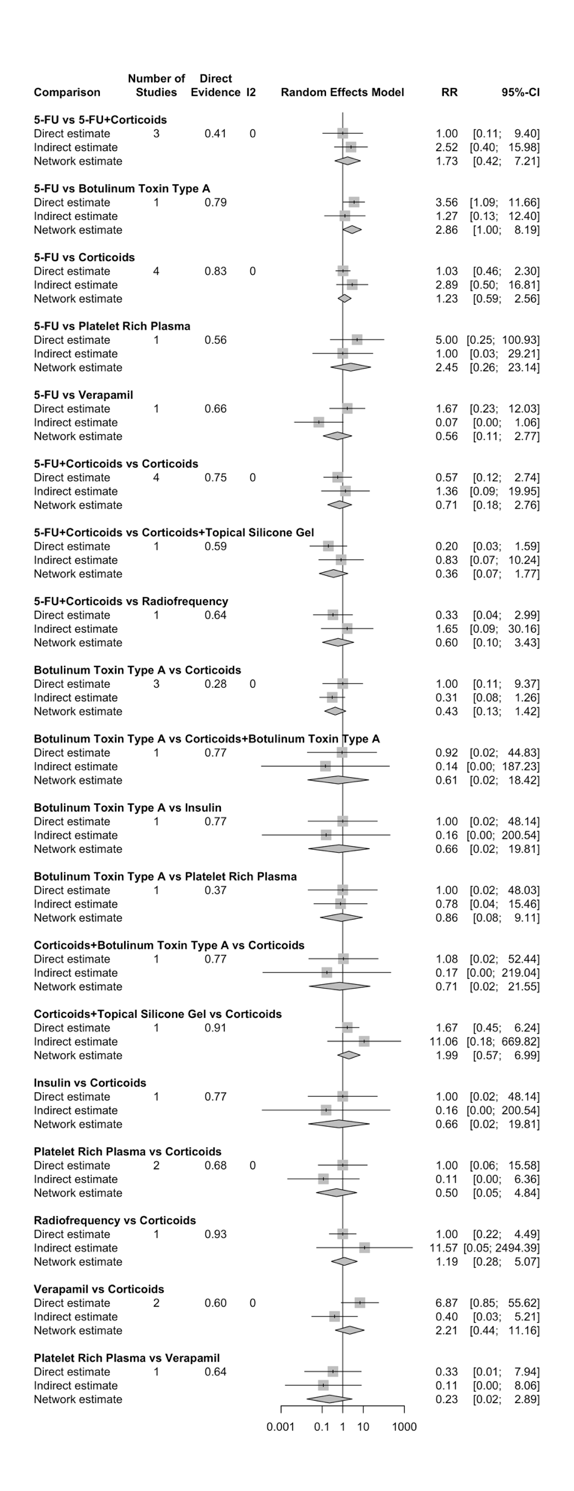


This node-split analysis compares direct, indirect, and network estimates of Relative Risk (RR) with 95% confidence intervals (CI) for recurrence in the treatment of keloid scars. Direct estimates (squares) come from head-to-head studies, indirect estimates are inferred through network meta-analysis, and network estimates (diamonds) combine both. Differences between direct and indirect estimates indicate potential network inconsistency.

**Supplementary Figure 10. Rank Probability Distributions for recurrence**


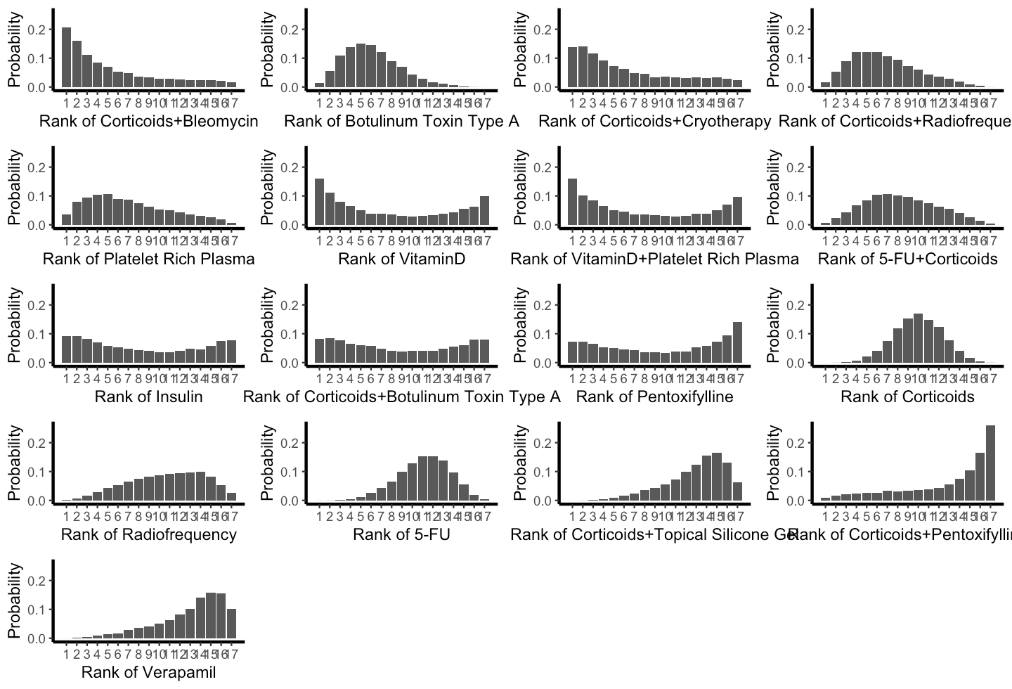


This figure presents the rank probability distributions of the included interventions for recurrence based on network meta-analysis. Each panel shows the probability of an intervention achieving a particular rank, with lower ranks indicating higher effectiveness. A left-skewed distribution suggests a higher likelihood of being among the top-ranked treatments, while a right-skewed distribution indicates a higher probability of being among the least effective.

**Supplementary Figure 11. Funnel Plot for Publication Bias in Network Meta-Analysis of recurrence**


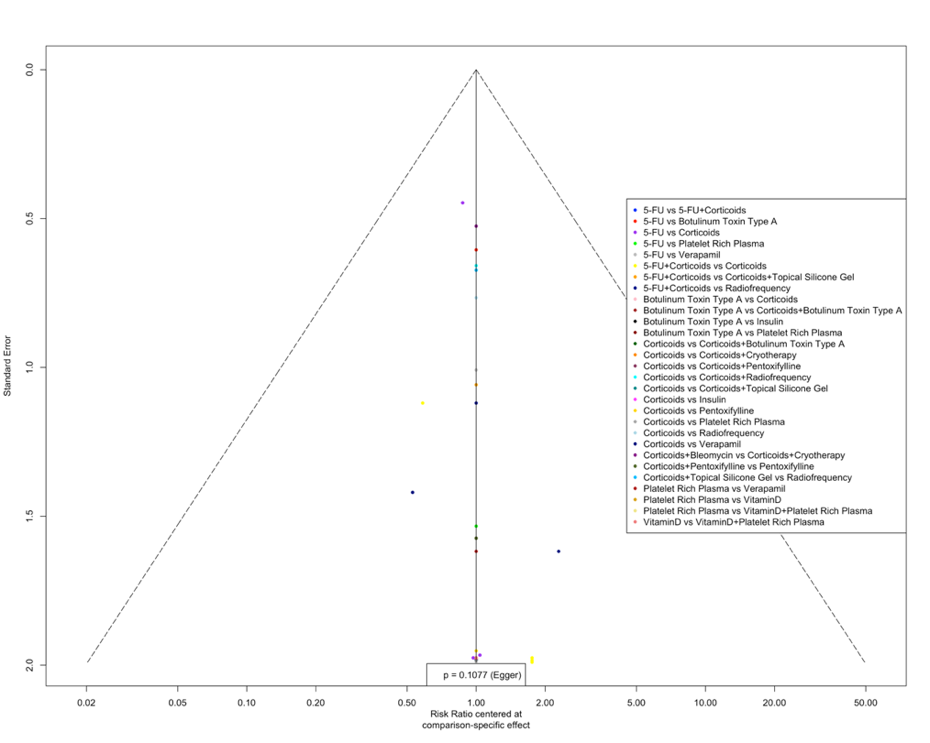


This funnel plot assesses small-study effects and potential publication bias in the network meta-analysis. The x-axis represents the Relative risk centered at the comparison-specific effect, while the y-axis shows the standard error. Each point represents a treatment comparison, color-coded according to the legend. The dashed lines indicate the 95% confidence region. The presence of symmetry suggests no publication bias, supported by Egger’s test (p = 0.10).

**Supplementary Figure 12. Direct and Indirect Evidence Proportions in Network Meta-Analysis for Adverse Events.**


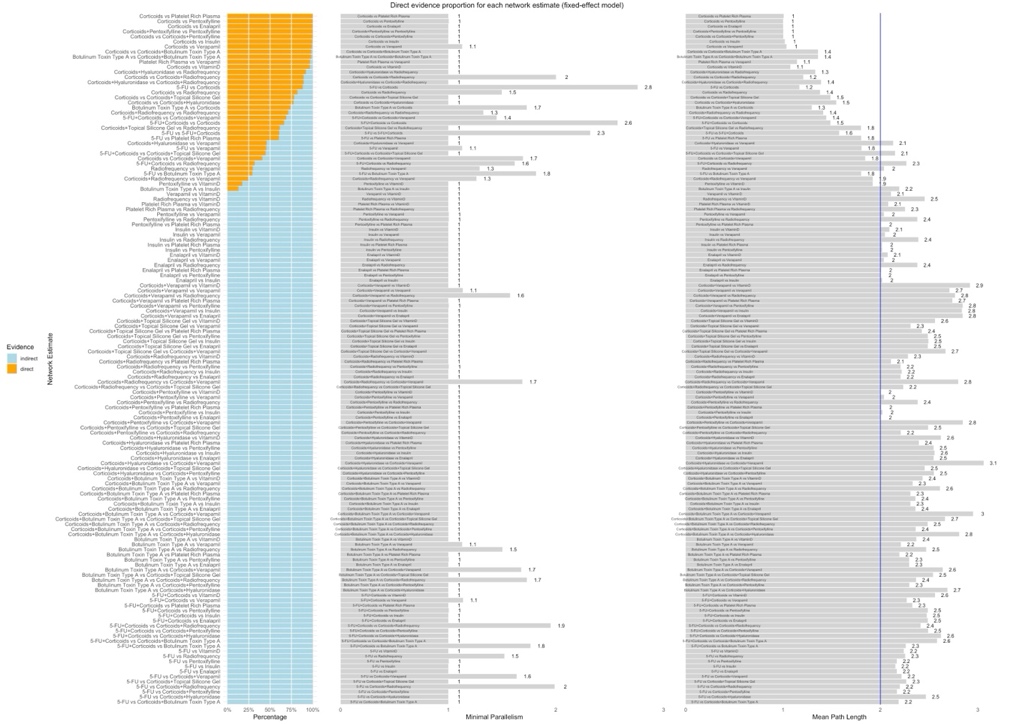


This figure presents the proportion of direct (orange) and indirect (blue) evidence for each network estimate, along with minimal parallelism and mean path length metrics in a fixed-effect model. The left panel shows the percentage of direct versus indirect contributions. The middle panel represents minimal parallelism values, indicating the minimum number of independent paths contributing to each comparison. The right panel displays the mean path length, reflecting the average number of steps connecting treatments in the network. A lower mean path length suggests stronger direct evidence.

**Supplementary Figure 13. Node-Split Analysis of Direct and Indirect Evidence for Adverse Events.**


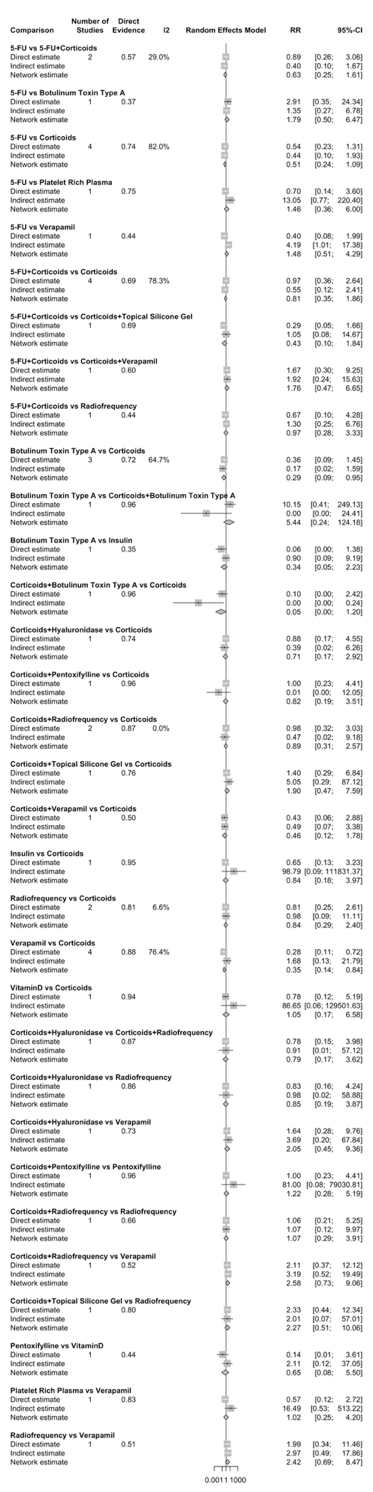


This node-split analysis compares direct, indirect, and network estimates of Relative Risk (RR) with 95% confidence intervals (CI) for adverse events in the treatment of keloid scars. Direct estimates (squares) come from head-to-head studies, indirect estimates are inferred through network meta-analysis, and network estimates (diamonds) combine both. Differences between direct and indirect estimates indicate potential network inconsistency.

### **Supplementary Figure 14. Rank Probability Distributions for adverse event.**


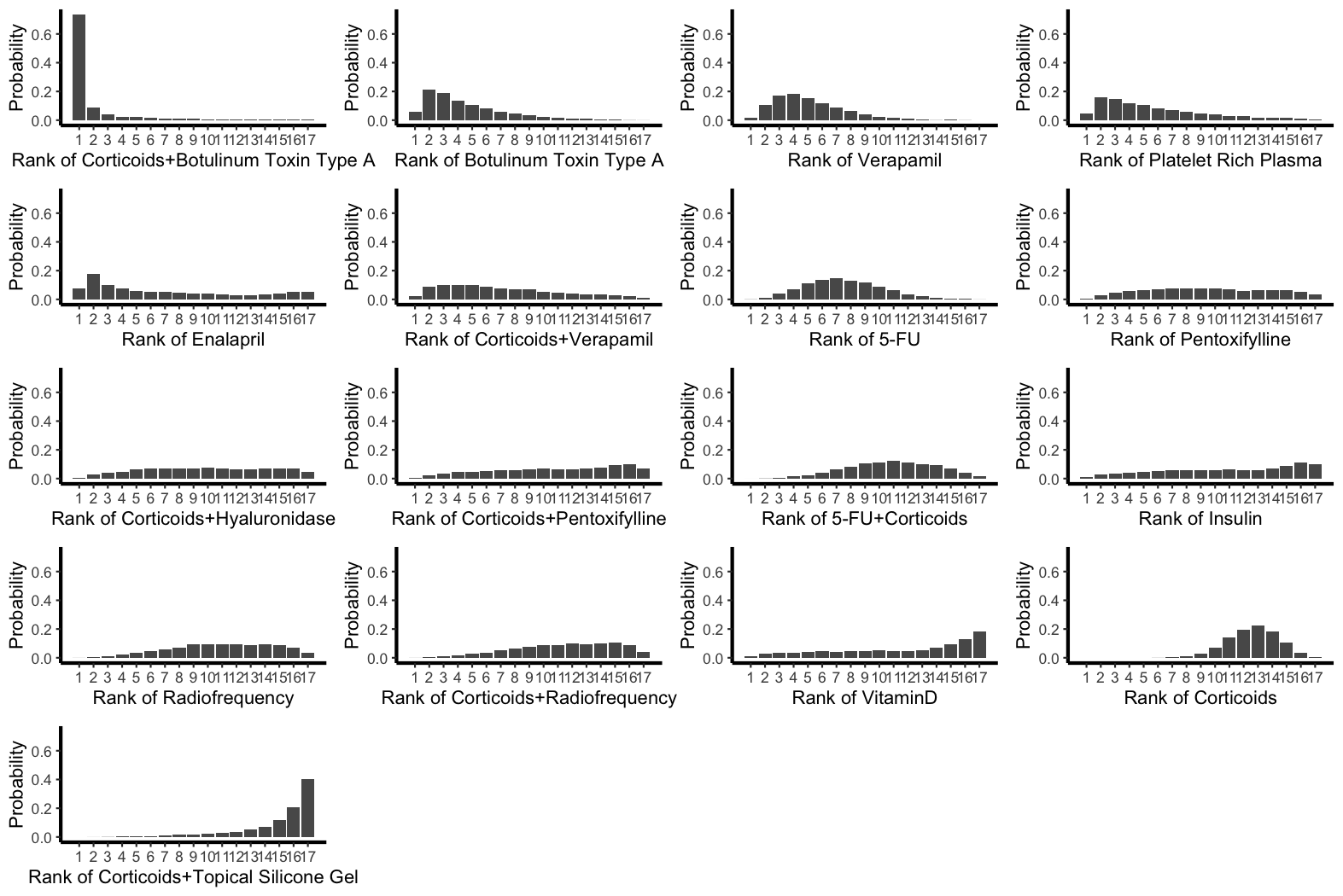


This figure presents the rank probability distributions of the included interventions for adverse events based on network meta-analysis. Each panel shows the probability of an intervention achieving a particular rank, with lower ranks indicating higher effectiveness. A left-skewed distribution suggests a higher likelihood of being among the top-ranked treatments, while a right-skewed distribution indicates a higher probability of being among the least effective.

**Supplementary Figure 15. Funnel Plot for Publication Bias in Network Meta-Analysis of Adverse Events.**


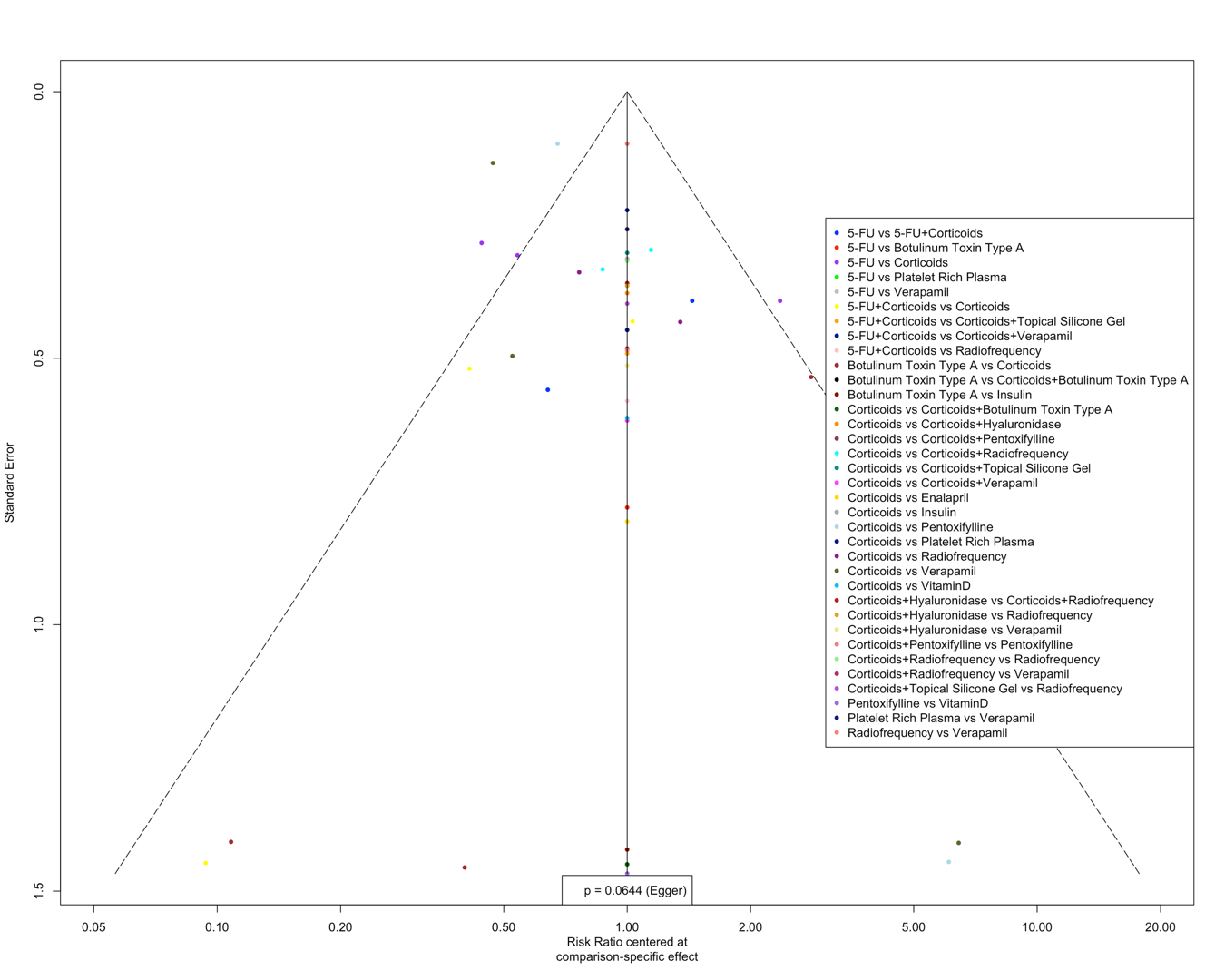


This funnel plot assesses small-study effects and potential publication bias in the network meta-analysis. The x-axis represents the Relative Risk centered at the comparison-specific effect, while the y-axis shows the standard error. Each point represents a treatment comparison, color-coded according to the legend. The dashed lines indicate the 95% confidence region. The presence of symmetry suggests no publication bias, supported by Egger’s test (p = 0.06).

**Supplementary Figure 16. Network Meta-Analysis of effectiveness with only low and some concerns risk of bias studies.**


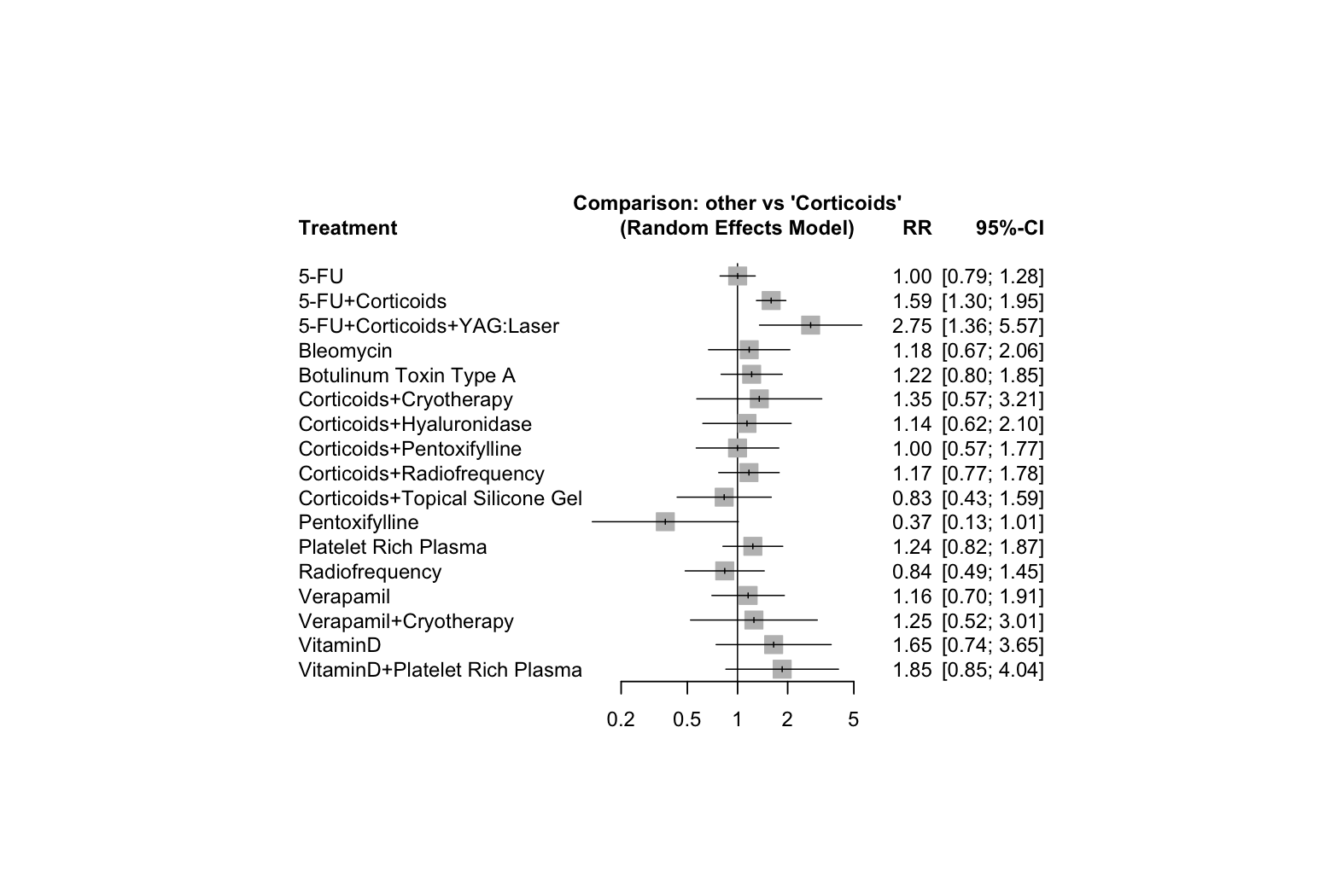


Forest plot showing standardized mean differences (SMD) with 95% confidence intervals for effectiveness of interventions versus corticoids. Positive RR favors the intervention. Squares represent effect sizes, and horizontal lines indicate confidence intervals.

**Supplementary Figure 17. Network Meta-Analysis of adverse events with only low and some concerns risk of bias studies.**


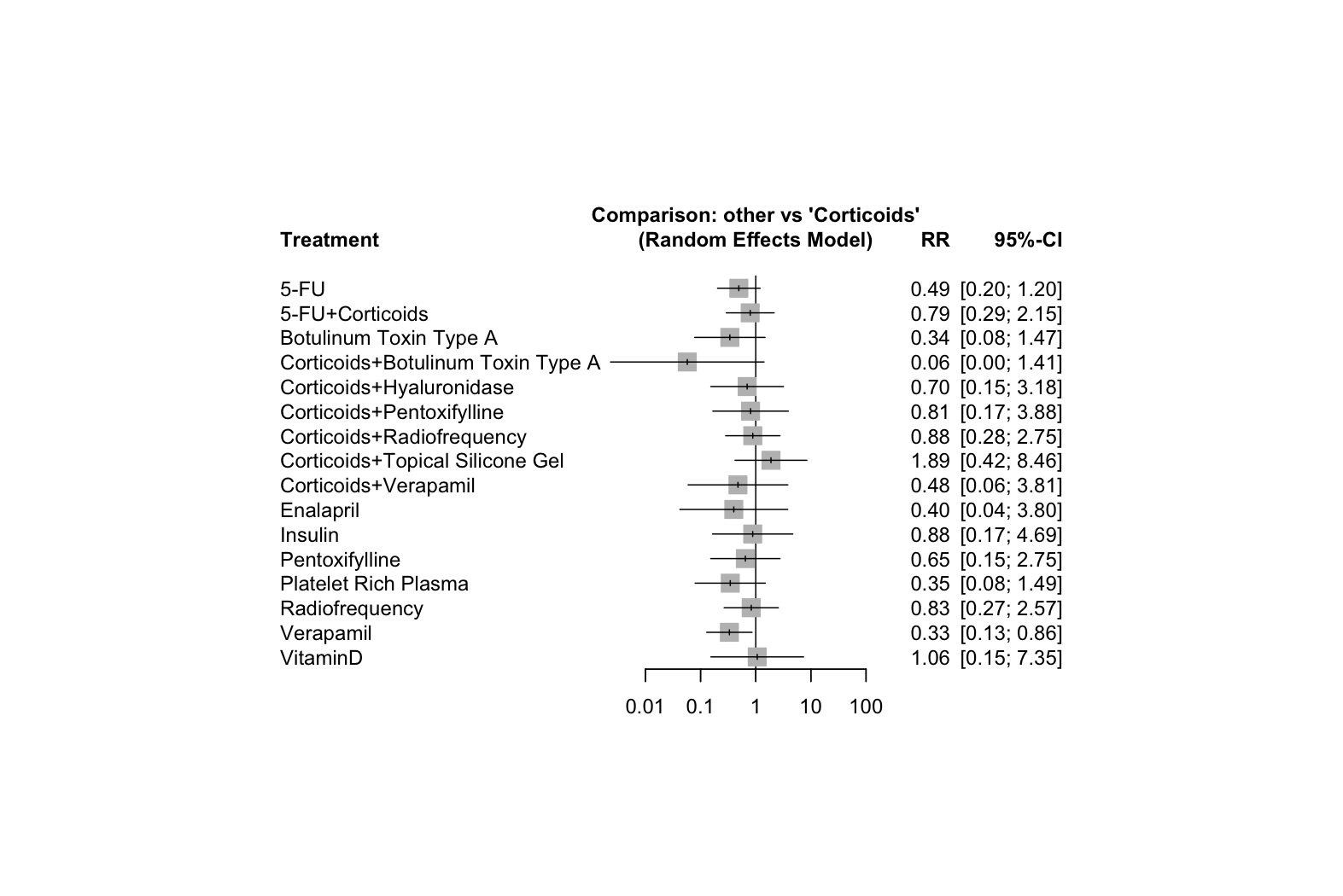


Forest plot showing standardized mean differences (SMD) with 95% confidence intervals for effectiveness of interventions versus corticoids. Negative RR favors the intervention. Squares represent effect sizes, and horizontal lines indicate confidence intervals.

**Tables**

**Supplementary Table 1. PubMed/MEDLINE search strategy and results**

| **Database** | PubMed/MEDLINE |
| --- | --- |
| **Date searched** | July 15, 2025 |
| **Search strategy** | keloid[Mesh] OR keloid*[tiab] OR “keloid scar”[tiab] OR pathologic scar*[tiab] OR fibrous scar*[tiab]) AND (intralesional therapy[tiab] OR intralesional injection*[tiab] OR IL injection*[tiab] OR steroid injection*[tiab] OR injection therapy[tiab] OR (injection[tiab] AND (triamcinolone[tiab] OR 5-fluorouracil[tiab] OR 5-FU[tiab] OR fluorouracil[tiab] OR bleomycin[tiab] OR verapamil[tiab] OR interferon[tiab] OR “botulinum toxin”[tiab] OR mitomycin[tiab] OR insulin[tiab] OR cryotherapy[tiab] OR cryosurgery[tiab] OR 5fu[tiab])) |
| **Results** | 491 |

**Supplementary Table 2. Embase search strategy and results**

| **Database** | Embase |
| --- | --- |
| **Date searched** | July 15, 2025 |
| **Search strategy** | (keloid/exp OR keloid*:ti,ab OR “keloid scar”:ti,ab) AND (intralesional therapy:ti,ab OR intralesional injection*:ti,ab OR IL injection*:ti,ab OR steroid injection*:ti,ab OR injection therapy:ti,ab OR (injection:ti,ab AND (triamcinolone:ti,ab OR 5-fluorouracil:ti,ab OR 5-FU:ti,ab OR fluorouracil:ti,abOR bleomycin:ti,ab OR verapamil:ti,ab OR interferon:ti,ab OR “botulinum toxin”:ti,ab OR mitomycin:ti,ab OR insulin:ti,ab OR cryotherapy:ti,ab OR cryosurgery:ti,ab OR 5fu:ti,ab))) |
| **Results** | 401 |

**Supplementary Table 3. Web of Science search strategy and results**

| **Database** | Web of Science |
| --- | --- |
| **Date searched** | July 15, 2025 |
| **Search strategy** | TS=(keloid* OR “keloid scar”* OR pathologic scar* OR fibrous scar*) AND TS=(intralesional therapy OR intralesional injection* OR IL injection* OR steroid injection* OR injection therapy OR (injection AND (triamcinolone OR 5-fluorouracil OR 5-FU OR fluorouracil OR bleomycin OR verapamil OR interferon OR “botulinum toxin” OR mitomycin OR insulin OR cryotherapy OR cryosurgery OR 5fu))) |
| **Results** | 975 |

**Supplementary Table 4. Cochrane CENTRAL search strategy and results**

| **Database** | Cochrane | | |
| --- | --- | --- | --- |
| **Date searched** | July 15, 2025 | | |
| **Search strategy No.** | #1 | keloid*:ti,ab,kw OR keloid scar*:ti,ab,kw OR pathologic scar*:ti,ab,kw OR fibrous scar*:ti,ab,kw | 958 |
|  | #2 | MeSH descriptor: [Keloid] explode all trees | 244 |
|  | #3 | intralesional therapy:ti,ab,kw OR intralesional injection*:ti,ab,kw OR IL injection*:ti,ab,kw OR steroid injection*:ti,ab,kw OR injection therapy:ti,ab,kw | 46983 |
|  | #4 | injection:ti,ab,kw | 97140 |
|  | #5 | triamcinolone:ti,ab,kw OR "5 fluorouracil":ti,ab,kw OR "5 FU":ti,ab,kw OR fluorouracil:ti,ab,kw OR bleomycin:ti,ab,kw OR verapamil:ti,ab,kw OR interferon:ti,ab,kw OR "botulinum toxin":ti,ab,kw OR mitomycin:ti,ab,kw OR "mitomycin C":ti,ab,kw OR insulin:ti,ab,kw OR cryotherapy:ti,ab,kw OR cryosurgery:ti,ab,kw OR 5fu:ti,ab,kw | 127579 |
|  | #6 | #1 OR #2 | 958 |
|  | #7 | #4 AND #5 | 11973 |
|  | #8 | #7 OR #3 | 51426 |
|  | #9 | #6 AND #8 | 317 |
| **Results** | 317 | | |

**Supplementary Table 5. CINAHL search strategy and results**

| **Database** | CINAHL |
| --- | --- |
| **Date searched** | July 15, 2025 |
| **Search strategy** | (TI(keloid*) OR AB(keloid*) OR TI(keloid scar*) OR AB(keloid scar*) OR TI(pathologic scar*) OR AB(pathologic scar*) OR TI(fibrous scar*) OR AB(fibrous scar*)) AND (TI(intralesional therapy) OR AB(intralesional therapy) OR TI(intralesional injection*) OR AB(intralesional injection*) OR TI(IL injection*) OR AB(IL injection*) OR TI(steroid injection*) OR AB(steroid injection*) OR TI(injection therapy) OR AB(injection therapy) OR ((TI(injection) OR AB(injection)) AND (TI(triamcinolone) OR AB(triamcinolone) OR TI(5-fluorouracil) OR AB(5-fluorouracil) OR TI(5-FU) OR AB(5-FU) OR TI(fluorouracil) OR AB(fluorouracil) OR TI(bleomycin) OR AB(bleomycin) OR TI(verapamil) OR AB(verapamil) OR TI(interferon) OR AB(interferon) OR TI(botulinum toxin) OR AB(botulinum toxin) OR TI(mitomycin) OR AB(mitomycin) OR TI(mitomycin C) OR AB(mitomycin C) OR TI(insulin) OR AB(insulin) OR TI(cryotherapy) OR AB(cryotherapy) OR TI(cryosurgery) OR AB(cryosurgery) OR TI(5fu) OR AB(5fu))) |
| **Results** | 74 |

**Supplementary Table 6. Scopus search strategy and results**

| **Database** | Scopus |
| --- | --- |
| **Date searched** | July 15, 2025 |
| **Search strategy** | (TITLE-ABS(keloid* OR “keloid scar”* OR pathologic scar* OR fibrous scar*)) AND (TITLE-ABS(intralesional therapy OR intralesional injection* OR IL injection* OR steroid injection* OR injection therapy OR (injection AND (triamcinolone OR 5-fluorouracil OR 5-FU OR fluorouracil OR bleomycin OR verapamil OR interferon OR botulinum toxin OR mitomycin OR mitomycin C OR insulin OR cryotherapy OR cryosurgery OR 5fu))) |
| **Results** | 174 |

**Supplementary Table 7. Google Scholar search strategy and results**

| **Database** | Google Scholar |
| --- | --- |
| **Date searched** | July 15, 2025 |
| **Search strategy** | (keloid OR “keloid scar” OR “pathologic scar” OR “fibrous scar”) AND (“intralesional therapy” OR “intralesional injection” OR “steroid injection” OR “injection therapy” OR (injection AND (triamcinolone OR “5-fluorouracil” OR “5-FU” OR fluorouracil OR bleomycin OR “botulinum toxin” OR insulin OR cryotherapy OR 5fu))) |
| **Results** | 200 |

**Supplementary Table 8: Comparison of Direct and Indirect Evidence in Network Meta-Analysis of Effectiveness**.

| **Comparison** | **k** | **Prop** | **NMA** | **95%-CI** | **Direct** | **95%-CI** | **Indirect** | **95%-CI** | **RoR** | **95%-CI** | **z** | **p-value** |
| --- | --- | --- | --- | --- | --- | --- | --- | --- | --- | --- | --- | --- |
| 5-FU vs 5-FU+Corticoids | 3 | 0.52 | 0.6677 | [0.5263; 0.8471] | 0.7739 | [0.5555; 1.0783] | 0.5708 | [0.4056; 0.8034] | 1.3558 | [0.8421; 2.1828] | 1.25 | 0.2103 |
| 5-FU vs Botulinum Toxin Type A | 1 | 0.33 | 0.7854 | [0.5411; 1.1399] | 0.6116 | [0.3208; 1.1660] | 0.8900 | [0.5639; 1.4046] | 0.6872 | [0.3118; 1.5146] | -0.93 | 0.3522 |
| 5-FU vs Corticoids | 7 | 0.71 | 1.0584 | [0.8589; 1.3042] | 1.0164 | [0.7928; 1.3032] | 1.1665 | [0.7934; 1.7149] | 0.8714 | [0.5509; 1.3784] | -0.59 | 0.5562 |
| 5-FU vs Platelet Rich Plasma | 1 | 0.44 | 0.8186 | [0.5413; 1.2379] | 0.7586 | [0.4077; 1.4118] | 0.8698 | [0.4997; 1.5140] | 0.8722 | [0.3794; 2.0052] | -0.32 | 0.7475 |
| 5-FU vs Verapamil | 1 | 0.54 | 0.8928 | [0.5709; 1.3962] | 0.6875 | [0.3732; 1.2666] | 1.2066 | [0.6261; 2.3252] | 0.5698 | [0.2325; 1.3965] | -1.23 | 0.2188 |
| 5-FU+Corticoids vs 5-FU+Corticoids+ YAG:Laser | 1 | 0.88 | 0.5800 | [0.2937; 1.1455] | 0.6875 | [0.3329; 1.4199] | 0.1657 | [0.0231; 1.1867] | 4.1490 | [0.5090; 33.8175] | 1.33 | 0.1838 |
| 5-FU+Corticoids vs Corticoids | 10 | 0.82 | 1.5851 | [1.3071; 1.9222] | 1.6962 | [1.3712; 2.0981] | 1.1588 | [0.7335; 1.8308] | 1.4637 | [0.8839; 2.4239] | 1.48 | 0.1388 |
| 5-FU+Corticoids vs Corticoids+ Topical Silicone Gel | 1 | 0.86 | 1.9061 | [1.0072; 3.6072] | 1.6667 | [0.8362; 3.3217] | 4.2190 | [0.7879; 22.5899] | 0.3950 | [0.0644; 2.4238] | -1.00 | 0.3157 |
| 5-FU+Corticoids vs Radiofrequency | 1 | 0.71 | 1.8845 | [1.0992; 3.2309] | 1.3333 | [0.7020; 2.5326] | 4.3258 | [1.6006; 11.6906] | 0.3082 | [0.0944; 1.0063] | -1.95 | 0.0512 |
| 5-FU+ Corticoids+YAG:Laser vs Corticoids | 1 | 0.33 | 2.7329 | [1.3617; 5.4848] | 5.3333 | [1.5949; 17.8349] | 1.9573 | [0.8341; 4.5929] | 2.7249 | [0.6215; 11.9477] | 1.33 | 0.1838 |
| Botulinum Toxin Type A vs Corticoids | 2 | 0.62 | 1.3476 | [0.9449; 1.9221] | 1.3160 | [0.8371; 2.0688] | 1.4001 | [0.7894; 2.4831] | 0.9399 | [0.4529; 1.9505] | -0.17 | 0.8679 |
| Botulinum Toxin Type A vs Platelet Rich Plasma | 1 | 0.56 | 1.0423 | [0.6749; 1.6097] | 1.0000 | [0.5589; 1.7893] | 1.0983 | [0.5713; 2.1114] | 0.9105 | [0.3795; 2.1843] | -0.21 | 0.8337 |
| Corticoids+ Cryotherapy vs Corticoids | 1 | 0.71 | 1.3513 | [0.8533; 2.1398] | 1.3319 | [0.7708; 2.3012] | 1.3991 | [0.5988; 3.2693] | 0.9519 | [0.3468; 2.6126] | -0.10 | 0.9238 |
| Corticoids+ Hyaluronidase vs Corticoids | 1 | 0.84 | 1.1342 | [0.6221; 2.0679] | 0.9200 | [0.4768; 1.7750] | 3.2747 | [0.7461; 14.3725] | 0.2809 | [0.0557; 1.4176] | -1.54 | 0.1242 |
| Corticoids+ Radiofrequency vs Corticoids | 2 | 0.97 | 1.1674 | [0.7752; 1.7580] | 1.0435 | [0.6891; 1.5803] | 68.4846 | [5.6187; 834.7351] | 0.0152 | [0.0012; 0.1922] | -3.24 | 0.0012 |
| Corticoids+ Topical Silicone Gel vs Corticoids | 1 | 0.74 | 0.8316 | [0.4378; 1.5796] | 0.8571 | [0.4061; 1.8091] | 0.7637 | [0.2182; 2.6734] | 1.1223 | [0.2610; 4.8264] | 0.16 | 0.8768 |
| Platelet Rich Plasma vs Corticoids | 2 | 0.70 | 1.2929 | [0.8704; 1.9205] | 1.3210 | [0.8227; 2.1210] | 1.2302 | [0.5985; 2.5286] | 1.0738 | [0.4534; 2.5431] | 0.16 | 0.8714 |
| Radiofrequency vs Corticoids | 2 | 0.76 | 0.8411 | [0.4909; 1.4411] | 0.7175 | [0.3874; 1.3292] | 1.4019 | [0.4641; 4.2345] | 0.5119 | [0.1444; 1.8149] | -1.04 | 0.2997 |
| Verapamil vs Corticoids | 2 | 0.58 | 1.1855 | [0.7728; 1.8185] | 0.9366 | [0.5350; 1.6397] | 1.6498 | [0.8499; 3.2022] | 0.5677 | [0.2383; 1.3525] | -1.28 | 0.2012 |
| Corticoids+ Cryotherapy vs Verapamil | 1 | 0.53 | 1.1398 | [0.6886; 1.8869] | 1.1667 | [0.5831; 2.3344] | 1.1106 | [0.5332; 2.3130] | 1.0505 | [0.3828; 2.8833] | 0.10 | 0.9238 |
| Corticoids+ Cryotherapy vs Verapamil+ Cryotherapy | 1 | 0.90 | 1.0656 | [0.5617; 2.0216] | 1.0769 | [0.5480; 2.1165] | 0.9712 | [0.1306; 7.2227] | 1.1089 | [0.1335; 9.2122] | 0.10 | 0.9238 |
| Corticoids+ Hyaluronidase vs Corticoids+ Radiofrequency | 1 | 0.86 | 0.9716 | [0.5281; 1.7874] | 0.9200 | [0.4768; 1.7750] | 1.3594 | [0.2661; 6.9432] | 0.6768 | [0.1166; 3.9266] | -0.44 | 0.6634 |
| Corticoids+ Hyaluronidase vs Radiofrequency | 1 | 0.36 | 1.3485 | [0.6220; 2.9236] | 4.8875 | [1.3396; 17.8324] | 0.6589 | [0.2509; 1.7299] | 7.4182 | [1.4759; 37.2856] | 2.43 | 0.0150 |
| Corticoids+ Hyaluronidase vs Verapamil | 1 | 0.07 | 0.9568 | [0.4598; 1.9911] | 21.5625 | [1.3226; 351.5264] | 0.7597 | [0.3555; 1.6237] | 28.3829 | [1.5730; 512.1425] | 2.27 | 0.0234 |
| Corticoids+ Radiofrequency vs Radiofrequency | 1 | 0.26 | 1.3879 | [0.7223; 2.6671] | 5.3125 | [1.4712; 19.1832] | 0.8687 | [0.4068; 1.8550] | 6.1156 | [1.3764; 27.1721] | 2.38 | 0.0173 |
| Corticoids+ Radiofrequency vs Verapamil | 1 | 0.04 | 0.9848 | [0.5465; 1.7744] | 23.4375 | [1.4446; 380.2677] | 0.8492 | [0.4649; 1.5510] | 27.6009 | [1.5951; 477.5946] | 2.28 | 0.0225 |
| Corticoids+ Topical Silicone Gel vs Radiofrequency | 1 | 0.95 | 0.9887 | [0.4839; 2.0201] | 0.8000 | [0.3838; 1.6677] | 40.5189 | [1.8697; 878.1048] | 0.0197 | [0.0008; 0.4665] | -2.43 | 0.0150 |
| Platelet Rich Plasma vs Verapamil | 1 | 0.75 | 1.0906 | [0.6623; 1.7961] | 0.9062 | [0.5097; 1.6113] | 1.9094 | [0.7019; 5.1938] | 0.4746 | [0.1496; 1.5056] | -1.27 | 0.2058 |
| Radiofrequency vs Verapamil | 1 | 0.05 | 0.7095 | [0.3580; 1.4063] | 4.4118 | [0.2193; 88.7336] | 0.6419 | [0.3179; 1.2960] | 6.8732 | [0.3151; 149.9267] | 1.23 | 0.2203 |
| Verapamil vs Verapamil+ Cryotherapy | 1 | 0.86 | 0.9349 | [0.4837; 1.8069] | 0.9231 | [0.4544; 1.8750] | 1.0140 | [0.1694; 6.0716] | 0.9103 | [0.1328; 6.2397] | -0.10 | 0.9238 |

**Supplementary Table 9. Comparison of Direct and Indirect Evidence in Network Meta-Analysis of recurrence.**

| **Comparison** | **k** | **Prop** | **NMA** | **95%-CI** | **Direct** | **95%-CI** | **Indirect** | **95%-CI** | **RoR** | **95%-CI** | **z** | **p-value** |
| --- | --- | --- | --- | --- | --- | --- | --- | --- | --- | --- | --- | --- |
| 5-FU vs 5-FU+Corticoids | 3 | 0.41 | 1.7317 | [0.4162; 7.2059] | 1.0000 | [0.1064; 9.3958] | 2.5167 | [0.3963; 15.9821] | 0.3973 | [0.0218; 7.2535] | -0.62 | 0.5334 |
| 5-FU vs Botulinum Toxin Type A | 1 | 0.79 | 2.8590 | [0.9983; 8.1872] | 3.5619 | [1.0878; 11.6634] | 1.2705 | [0.1302; 12.4007] | 2.8036 | [0.2149; 36.5811] | 0.79 | 0.4315 |
| 5-FU vs Corticoids | 4 | 0.83 | 1.2313 | [0.5926; 2.5586] | 1.0299 | [0.4609; 2.3017] | 2.8945 | [0.4983; 16.8121] | 0.3558 | [0.0514; 2.4622] | -1.05 | 0.2951 |
| 5-FU vs Platelet Rich Plasma | 1 | 0.56 | 2.4547 | [0.2604; 23.1425] | 5.0000 | [0.2477; 100.9273] | 1.0016 | [0.0343; 29.2111] | 4.9918 | [0.0545; 457.2029] | 0.70 | 0.4854 |
| 5-FU vs Verapamil | 1 | 0.66 | 0.5577 | [0.1124; 2.7676] | 1.6667 | [0.2308; 12.0330] | 0.0688 | [0.0045; 1.0580] | 24.2402 | [0.8307; 707.3364] | 1.85 | 0.0640 |
| 5-FU+Corticoids vs Corticoids | 4 | 0.75 | 0.7110 | [0.1834; 2.7560] | 0.5703 | [0.1188; 2.7380] | 1.3580 | [0.0925; 19.9451] | 0.4200 | [0.0187; 9.4296] | -0.55 | 0.5847 |
| 5-FU+Corticoids vs Corticoids+ Topical Silicone Gel | 1 | 0.59 | 0.3573 | [0.0723; 1.7668] | 0.2000 | [0.0251; 1.5917] | 0.8344 | [0.0680; 10.2433] | 0.2397 | [0.0093; 6.2088] | -0.86 | 0.3896 |
| 5-FU+Corticoids vs Radiofrequency | 1 | 0.64 | 0.5955 | [0.1034; 3.4311] | 0.3333 | [0.0371; 2.9910] | 1.6484 | [0.0901; 30.1554] | 0.2022 | [0.0053; 7.7164] | -0.86 | 0.3896 |
| Botulinum Toxin Type A vs Corticoids | 3 | 0.28 | 0.4307 | [0.1309; 1.4166] | 1.0000 | [0.1067; 9.3740] | 0.3088 | [0.0757; 1.2601] | 3.2382 | [0.2304; 45.5170] | 0.87 | 0.3836 |
| Botulinum Toxin Type A vs Corticoids+ Botulinum Toxin Type A | 1 | 0.77 | 0.6067 | [0.0200; 18.4206] | 0.9245 | [0.0191; 44.8254] | 0.1442 | [0.0001; 187.2272] | 6.4123 | [0.0018; 22257.5667] | 0.45 | 0.6551 |
| Botulinum Toxin Type A vs Insulin | 1 | 0.77 | 0.6563 | [0.0217; 19.8115] | 1.0000 | [0.0208; 48.1384] | 0.1556 | [0.0001; 200.5412] | 6.4272 | [0.0019; 22088.2913] | 0.45 | 0.6543 |
| Botulinum Toxin Type A vs Platelet Rich Plasma | 1 | 0.37 | 0.8586 | [0.0809; 9.1109] | 1.0000 | [0.0208; 48.0301] | 0.7844 | [0.0398; 15.4560] | 1.2749 | [0.0096; 168.8820] | 0.10 | 0.9224 |
| Corticoids+Botulinum Toxin Type A vs Corticoids | 1 | 0.77 | 0.7098 | [0.0234; 21.5509] | 1.0816 | [0.0223; 52.4426] | 0.1687 | [0.0001; 219.0426] | 6.4123 | [0.0018; 22257.5667] | 0.45 | 0.6551 |
| Corticoids+Topical Silicone Gel vs Corticoids | 1 | 0.91 | 1.9900 | [0.5666; 6.9896] | 1.6667 | [0.4454; 6.2368] | 11.0560 | [0.1825; 669.8165] | 0.1507 | [0.0020; 11.2326] | -0.86 | 0.3896 |
| Insulin vs Corticoids | 1 | 0.77 | 0.6563 | [0.0217; 19.8115] | 1.0000 | [0.0208; 48.1384] | 0.1556 | [0.0001; 200.5412] | 6.4272 | [0.0019; 22088.2913] | 0.45 | 0.6543 |
| Platelet Rich Plasma vs Corticoids | 2 | 0.68 | 0.5016 | [0.0520; 4.8384] | 1.0000 | [0.0642; 15.5830] | 0.1149 | [0.0021; 6.3603] | 8.7051 | [0.0672; 1127.0991] | 0.87 | 0.3832 |
| Radiofrequency vs Corticoids | 1 | 0.93 | 1.1940 | [0.2812; 5.0690] | 1.0000 | [0.2229; 4.4872] | 11.5729 | [0.0537; 2494.3891] | 0.0864 | [0.0003; 22.8795] | -0.86 | 0.3896 |
| Verapamil vs Corticoids | 2 | 0.60 | 2.2077 | [0.4368; 11.1573] | 6.8685 | [0.8482; 55.6175] | 0.4022 | [0.0310; 5.2123] | 17.0764 | [0.6253; 466.3124] | 1.68 | 0.0926 |
| Platelet Rich Plasma vs Verapamil | 1 | 0.64 | 0.2272 | [0.0179; 2.8918] | 0.3333 | [0.0140; 7.9424] | 0.1137 | [0.0016; 8.0597] | 2.9308 | [0.0145; 593.7282] | 0.40 | 0.6915 |

**Supplementary Table 10. Comparison of Direct and Indirect Evidence in Network Meta-Analysis of adverse events.**

| **Comparison** | **k** | **Prop** | **NMA** | **95%-CI** | **Direct** | **95%-CI** | **Indirect** | **95%-CI** | **RoR** | **95%-CI** | **z** | **p-value** |
| --- | --- | --- | --- | --- | --- | --- | --- | --- | --- | --- | --- | --- |
| 5-FU vs 5-FU+Corticoids | 2 | 0.57 | 0.6347 | [0.2501; 1.6109] | 0.8925 | [0.2605; 3.0581] | 0.4026 | [0.0970; 1.6714] | 2.2167 | [0.3375; 14.5602] | 0.83 | 0.4072 |
| 5-FU vs Botulinum Toxin Type A | 1 | 0.37 | 1.7908 | [0.4959; 6.4674] | 2.9143 | [0.3489; 24.3425] | 1.3520 | [0.2695; 6.7820] | 2.1555 | [0.1499; 30.9927] | 0.56 | 0.5723 |
| 5-FU vs Corticoids | 4 | 0.74 | 0.5146 | [0.2424; 1.0927] | 0.5448 | [0.2274; 1.3053] | 0.4366 | [0.0990; 1.9260] | 1.2477 | [0.2229; 6.9840] | 0.25 | 0.8011 |
| 5-FU vs Platelet Rich Plasma | 1 | 0.75 | 1.4602 | [0.3552; 6.0020] | 0.7037 | [0.1376; 3.5995] | 13.0455 | [0.7722; 220.4005] | 0.0539 | [0.0021; 1.4113] | -1.75 | 0.0796 |
| 5-FU vs Verapamil | 1 | 0.44 | 1.4849 | [0.5135; 4.2943] | 0.4043 | [0.0821; 1.9916] | 4.1873 | [1.0086; 17.3839] | 0.0965 | [0.0114; 0.8186] | -2.14 | 0.0321 |
| 5-FU+Corticoids vs Corticoids | 4 | 0.69 | 0.8108 | [0.3540; 1.8568] | 0.9699 | [0.3569; 2.6359] | 0.5472 | [0.1245; 2.4059] | 1.7723 | [0.2969; 10.5808] | 0.63 | 0.5301 |
| 5-FU+Corticoids vs Corticoids+Topical Silicone Gel | 1 | 0.69 | 0.4272 | [0.0989; 1.8449] | 0.2857 | [0.0492; 1.6593] | 1.0529 | [0.0755; 14.6736] | 0.2714 | [0.0114; 6.4465] | -0.81 | 0.4197 |
| 5-FU+Corticoids vs Corticoids+Verapamil | 1 | 0.60 | 1.7638 | [0.4680; 6.6475] | 1.6667 | [0.3004; 9.2456] | 1.9199 | [0.2358; 15.6317] | 0.8681 | [0.0579; 13.0197] | -0.10 | 0.9184 |
| 5-FU+Corticoids vs Radiofrequency | 1 | 0.44 | 0.9678 | [0.2816; 3.3263] | 0.6667 | [0.1038; 4.2835] | 1.2978 | [0.2491; 6.7606] | 0.5137 | [0.0427; 6.1762] | -0.53 | 0.5996 |
| Botulinum Toxin Type A vs Corticoids | 3 | 0.72 | 0.2874 | [0.0870; 0.9493] | 0.3559 | [0.0872; 1.4532] | 0.1651 | [0.0172; 1.5890] | 2.1555 | [0.1499; 30.9927] | 0.56 | 0.5723 |
| Botulinum Toxin Type A vs Corticoids+Botulinum Toxin Type A | 1 | 0.96 | 5.4430 | [0.2386; 124.1805] | 10.1538 | [0.4138; 249.1289] | 0.0000 | [0.0000; 24.4084] | 1063102.4542 | [0.2952; 3.8287e+12] | 1.80 | 0.0716 |
| Botulinum Toxin Type A vs Insulin | 1 | 0.35 | 0.3437 | [0.0529; 2.2331] | 0.0588 | [0.0025; 1.3757] | 0.8984 | [0.0878; 9.1946] | 0.0655 | [0.0013; 3.2914] | -1.36 | 0.1726 |
| Corticoids+Botulinum Toxin Type A vs Corticoids | 1 | 0.96 | 0.0528 | [0.0023; 1.2045] | 0.0985 | [0.0040; 2.4164] | 0.0000 | [0.0000; 0.2367] | 1063102.4542 | [0.2952; 3.8287e+12] | 1.80 | 0.0716 |
| Corticoids+Hyaluronidase vs Corticoids | 1 | 0.74 | 0.7092 | [0.1720; 2.9246] | 0.8750 | [0.1684; 4.5471] | 0.3911 | [0.0244; 6.2645] | 2.2374 | [0.0888; 56.3551] | 0.49 | 0.6247 |
| Corticoids+Pentoxifylline vs Corticoids | 1 | 0.96 | 0.8226 | [0.1927; 3.5107] | 1.0000 | [0.2266; 4.4128] | 0.0123 | [0.0000; 12.0455] | 81.0000 | [0.0709; 92582.84] | 1.22 | 0.2213 |
| Corticoids+Radiofrequency vs Corticoids | 2 | 0.87 | 0.8930 | [0.3108; 2.5657] | 0.9797 | [0.3168; 3.0295] | 0.4702 | [0.0241; 9.1804] | 2.0837 | [0.0867; 50.0529] | 0.45 | 0.6508 |
| Corticoids+Topical Silicone Gel vs Corticoids | 1 | 0.76 | 1.8979 | [0.4745; 7.5907] | 1.4000 | [0.2864; 6.8444] | 5.0539 | [0.2932; 87.1233] | 0.2770 | [0.0106; 7.2128] | -0.77 | 0.4402 |
| Corticoids+Verapamil vs Corticoids | 1 | 0.50 | 0.4597 | [0.1187; 1.7801] | 0.4286 | [0.0638; 2.8809] | 0.4937 | [0.0721; 3.3815] | 0.8681 | [0.0579; 13.0197] | -0.10 | 0.9184 |
| Insulin vs Corticoids | 1 | 0.95 | 0.8362 | [0.1763; 3.9668] | 0.6538 | [0.1325; 3.2272] | 98.7876 | [0.0873; 111831.3651] | 0.0066 | [0.0000; 8.9610] | -1.36 | 0.1726 |

**Supplementary Table 11. Surface Under the Cumulative Ranking Curve (SUCRA) Scores for effectiveness in low and some concerns risk of bias studies.**

| **Intervention** | **SUCRA** |
| --- | --- |
| 5-FU | 0.3385 |
| 5-FU+Corticoids | 0.7847 |
| 5-FU+Corticoids+ YAG:Laser | 0.9496 |
| Bleomycin | 0.4989 |
| Botulinum Toxin Type A | 0.5366 |
| Corticoids | 0.3265 |
| Corticoids+Cryotherapy | 0.6022 |
| Corticoids+Hyaluronidase | 0.4714 |
| Corticoids+Pentoxifylline | 0.3634 |
| Corticoids+Radiofrequency | 0.5002 |
| Corticoids+Topical Silicone Gel | 0.2383 |
| Pentoxifylline | 0.0285 |
| Platelet Rich Plasma | 0.5552 |
| Radiofrequency | 0.2270 |
| Verapamil | 0.4876 |
| Verapamil+Cryotherapy | 0.5444 |
| VitaminD | 0.7400 |
| VitaminD+Platelet Rich Plasma | 0.8071 |

**Supplementary Table 12. League Table of Relative Risk (RR) and 95% Confidence Intervals for effectiveness in only low and some concerns risk of bias studies.**

| 5-FU | 0.79 [0.52; 1.19] | . | . | . | 0.92 [0.71; 1.21] | . | . | . | . | . | . | 0.76 [0.40; 1.43] | . | 0.69 [0.37; 1.28] | . | . | . |  |
| --- | --- | --- | --- | --- | --- | --- | --- | --- | --- | --- | --- | --- | --- | --- | --- | --- | --- | --- |
| 0.63 [0.48; 0.83] | 5-FU+Corticoids | 0.69 [0.33; 1.43] | . | . | 1.70 [1.37; 2.11] | . | . | . | . | 1.67 [0.83; 3.36] | . | . | 1.33 [0.69; 2.56] | . | . | . | . |  |
| 0.36 [0.17; 0.76] | 0.58 [0.29; 1.15] | 5-FU+Corticoids+YAG:Laser | . | . | 5.33 [1.59; 17.94] | . | . | . | . | . | . | . | . | . | . | . | . |  |
| 0.85 [0.46; 1.57] | 1.35 [0.74; 2.46] | 2.34 [0.95; 5.77] | Bleomycin | . | 1.18 [0.67; 2.06] | . | . | . | . | . | . | . | . | . | . | . | . |  |
| 0.82 [0.51; 1.32] | 1.31 [0.82; 2.09] | 2.26 [0.99; 5.15] | 0.97 [0.48; 1.95] | Botulinum Toxin Type A | 1.32 [0.83; 2.10] | . | . | . | . | . | . | 1.00 [0.55; 1.81] | . | . | . | . | . |  |
| 1.00 [0.79; 1.28] | 1.59 [1.30; 1.95] | 2.75 [1.36; 5.57] | 1.18 [0.67; 2.06] | 1.22 [0.80; 1.85] | Corticoids | . | 1.09 [0.56; 2.12] | 1.00 [0.57; 1.77] | 0.96 [0.63; 1.46] | 1.17 [0.55; 2.49] | 2.71 [0.99; 7.45] | 0.75 [0.47; 1.22] | 1.40 [0.75; 2.62] | 1.07 [0.61; 1.90] | . | . | . |  |
| 0.74 [0.31; 1.78] | 1.18 [0.49; 2.85] | 2.03 [0.67; 6.20] | 0.87 [0.31; 2.44] | 0.90 [0.35; 2.30] | 0.74 [0.31; 1.76] | Corticoids+Cryotherapy | . | . | . | . | . | . | . | 1.17 [0.58; 2.36] | 1.08 [0.54; 2.14] | . | . |  |
| 0.88 [0.46; 1.69] | 1.40 [0.74; 2.65] | 2.41 [0.95; 6.13] | 1.03 [0.45; 2.37] | 1.07 [0.51; 2.24] | 0.88 [0.48; 1.62] | 1.19 [0.41; 3.40] | Corticoids+Hyaluronidase | . | 0.92 [0.47; 1.79] | . | . | . | 4.89 [1.33; 17.93] | 21.56 [1.32; 352.45] | . | . | . |  |
| 1.00 [0.54; 1.86] | 1.59 [0.87; 2.92] | 2.75 [1.11; 6.82] | 1.18 [0.53; 2.62] | 1.22 [0.60; 2.47] | 1.00 [0.57; 1.77] | 1.35 [0.48; 3.80] | 1.14 [0.49; 2.63] | Corticoids+Pentoxifylline | . | . | 2.71 [0.99; 7.45] | . | . | . | . | . | . |  |
| 0.86 [0.53; 1.39] | 1.36 [0.86; 2.16] | 2.35 [1.03; 5.33] | 1.00 [0.50; 2.02] | 1.04 [0.57; 1.88] | 0.85 [0.56; 1.30] | 1.15 [0.44; 3.00] | 0.97 [0.52; 1.81] | 0.85 [0.42; 1.73] | Corticoids+Radiofrequency | . | . | . | 5.31 [1.46; 19.29] | 23.44 [1.44; 381.27] | . | . | . |  |
| 1.20 [0.61; 2.39] | 1.91 [1.00; 3.66] | 3.31 [1.29; 8.48] | 1.41 [0.60; 3.34] | 1.46 [0.67; 3.17] | 1.20 [0.63; 2.31] | 1.62 [0.55; 4.78] | 1.37 [0.57; 3.31] | 1.20 [0.51; 2.86] | 1.41 [0.65; 3.03] | Corticoids+Topical Silicone Gel | . | . | 0.80 [0.38; 1.68] | . | . | . | . |  |
| 2.72 [0.96; 7.68] | 4.32 [1.54; 12.10] | 7.46 [2.18; 25.59] | 3.19 [1.00; 10.13] | 3.30 [1.10; 9.86] | 2.71 [0.99; 7.45] | 3.67 [0.97; 13.85] | 3.09 [0.95; 10.07] | 2.71 [0.99; 7.45] | 3.18 [1.07; 9.48] | 2.26 [0.68; 7.50] | Pentoxifylline | . | . | . | . | . | . |  |
| 0.81 [0.52; 1.27] | 1.29 [0.82; 2.03] | 2.22 [0.98; 5.03] | 0.95 [0.47; 1.91] | 0.98 [0.61; 1.58] | 0.81 [0.54; 1.22] | 1.09 [0.45; 2.66] | 0.92 [0.44; 1.92] | 0.81 [0.40; 1.64] | 0.95 [0.53; 1.70] | 0.67 [0.31; 1.45] | 0.30 [0.10; 0.89] | Platelet Rich Plasma | . | 0.91 [0.50; 1.63] | . | 0.75 [0.38; 1.48] | 0.67 [0.34; 1.29] | |
| 1.20 [0.66; 2.15] | 1.90 [1.10; 3.29] | 3.28 [1.37; 7.87] | 1.40 [0.64; 3.07] | 1.45 [0.73; 2.89] | 1.19 [0.69; 2.06] | 1.61 [0.58; 4.47] | 1.36 [0.62; 2.98] | 1.19 [0.54; 2.63] | 1.40 [0.72; 2.71] | 0.99 [0.48; 2.05] | 0.44 [0.14; 1.39] | 1.48 [0.75; 2.92] | Radiofrequency | 4.41 [0.22; 88.95] | . | . | . |  |
| 0.87 [0.52; 1.45] | 1.37 [0.81; 2.34] | 2.37 [1.00; 5.62] | 1.02 [0.48; 2.16] | 1.05 [0.57; 1.95] | 0.86 [0.52; 1.43] | 1.17 [0.58; 2.36] | 0.98 [0.45; 2.16] | 0.86 [0.40; 1.85] | 1.01 [0.53; 1.93] | 0.72 [0.32; 1.63] | 0.32 [0.10; 0.98] | 1.07 [0.62; 1.84] | 0.72 [0.35; 1.51] | Verapamil | 0.92 [0.45; 1.89] | . | . |  |
| 0.80 [0.33; 1.94] | 1.27 [0.52; 3.10] | 2.19 [0.71; 6.73] | 0.94 [0.33; 2.65] | 0.97 [0.38; 2.50] | 0.80 [0.33; 1.91] | 1.08 [0.54; 2.14] | 0.91 [0.31; 2.63] | 0.80 [0.28; 2.27] | 0.93 [0.35; 2.46] | 0.66 [0.22; 1.97] | 0.29 [0.08; 1.12] | 0.99 [0.40; 2.43] | 0.67 [0.24; 1.87] | 0.92 [0.45; 1.89] | Verapamil+Cryotherapy | . | . |  |
| 0.61 [0.27; 1.37] | 0.97 [0.43; 2.19] | 1.67 [0.58; 4.82] | 0.71 [0.27; 1.89] | 0.74 [0.32; 1.69] | 0.61 [0.27; 1.34] | 0.82 [0.27; 2.51] | 0.69 [0.25; 1.88] | 0.61 [0.23; 1.61] | 0.71 [0.29; 1.74] | 0.50 [0.18; 1.41] | 0.22 [0.06; 0.81] | 0.75 [0.38; 1.48] | 0.51 [0.19; 1.33] | 0.70 [0.29; 1.68] | 0.76 [0.25; 2.35] | VitaminD | 0.89 [0.49; 1.61] | |
| 0.54 [0.24; 1.20] | 0.86 [0.39; 1.91] | 1.48 [0.52; 4.23] | 0.63 [0.24; 1.66] | 0.66 [0.29; 1.47] | 0.54 [0.25; 1.17] | 0.73 [0.24; 2.21] | 0.61 [0.23; 1.65] | 0.54 [0.21; 1.42] | 0.63 [0.26; 1.53] | 0.45 [0.16; 1.23] | 0.20 [0.06; 0.71] | 0.67 [0.34; 1.29] | 0.45 [0.17; 1.17] | 0.62 [0.27; 1.47] | 0.68 [0.22; 2.07] | 0.89 [0.49; 1.61] | VitaminD+Platelet Rich Plasma | |

**Supplementary Table 13. Surface Under the Cumulative Ranking Curve (SUCRA) Scores for adverse events in low and some concerns risk of bias studies.**

| **Intervention** | **SUCRA** |
| --- | --- |
| 5-FU | 0.6062 |
| 5-FU+Corticoids | 0.4058 |
| Botulinum Toxin Type A | 0.7032 |
| Corticoids | 0.2871 |
| Corticoids+Botulinum Toxin Type A | 0.9113 |
| Corticoids+Hyaluronidase | 0.4581 |
| Corticoids+Pentoxifylline | 0.4059 |
| Corticoids+Radiofrequency | 0.3632 |
| Corticoids+Topical Silicone Gel | 0.1493 |
| Corticoids+Verapamil | 0.5801 |
| Enalapril | 0.6141 |
| Insulin | 0.3799 |
| Pentoxifylline | 0.4830 |
| Platelet Rich Plasma | 0.7000 |
| Radiofrequency | 0.3840 |
| Verapamil | 0.7401 |
| Vitamin D | 0.3286 |

**Supplementary Table 14. League Table of Relative Risk (RR) and 95% Confidence Intervals for adverse events in only low and some concerns risk of bias studies.**

| 5-FU | 1.29 [0.22; 7.60] | . | 0.56 [0.22; 1.42] | . | . | . | . | . | . | . | . | . | 0.70 [0.12; 4.05] | . | 0.40 [0.07; 2.25] | . |
| --- | --- | --- | --- | --- | --- | --- | --- | --- | --- | --- | --- | --- | --- | --- | --- | --- |
| 0.62 [0.19; 2.08] | 5-FU+Corticoids | . | 0.96 [0.34; 2.77] | . | . | . | . | 0.29 [0.04; 1.85] | 1.67 [0.27; 10.35] | . | . | . | . | 0.67 [0.09; 4.76] | . | . |
| 1.45 [0.26; 8.09] | 2.34 [0.40; 13.80] | Botulinum Toxin Type A | 0.34 [0.08; 1.47] | 10.15 [0.39; 265.03] | . | . | . | . | . | . | 0.06 [0.00; 1.46] | . | . | . | . | . |
| 0.49 [0.20; 1.20] | 0.79 [0.29; 2.15] | 0.34 [0.08; 1.47] | Corticoids | 10.15 [0.39; 265.03] | 1.14 [0.20; 6.68] | 1.00 [0.20; 5.02] | 1.02 [0.30; 3.44] | 0.71 [0.13; 3.94] | . | 2.50 [0.26; 23.73] | 1.53 [0.27; 8.52] | 1.54 [0.36; 6.55] | 2.93 [0.56; 15.39] | 1.24 [0.35; 4.34] | 3.70 [1.35; 10.12] | 1.29 [0.17; 9.51] |
| 8.60 [0.31; 238.97] | 13.83 [0.48; 396.29] | 5.91 [0.24; 145.59] | 17.43 [0.71; 429.13] | Corticoids+Botulinum Toxin Type A | . | . | . | . | . | . | . | . | . | . | . | . |
| 0.71 [0.13; 3.96] | 1.14 [0.19; 6.67] | 0.49 [0.06; 4.01] | 1.43 [0.31; 6.52] | 0.08 [0.00; 2.84] | Corticoids+Hyaluronidase | . | 0.78 [0.14; 4.48] | . | . | . | . | . | . | 0.83 [0.14; 4.77] | 1.64 [0.25; 10.88] | . |
| 0.61 [0.10; 3.73] | 0.99 [0.15; 6.35] | 0.42 [0.05; 3.62] | 1.24 [0.26; 5.99] | 0.07 [0.00; 2.53] | 0.87 [0.10; 7.71] | Corticoids+Pentoxifylline | . | . | . | . | . | 1.00 [0.20; 5.02] | . | . | . | . |
| 0.56 [0.14; 2.30] | 0.90 [0.21; 3.92] | 0.38 [0.06; 2.45] | 1.13 [0.36; 3.52] | 0.06 [0.00; 1.94] | 0.79 [0.16; 4.02] | 0.91 [0.13; 6.33] | Corticoids+Radiofrequency | . | . | . | . | . | . | 1.06 [0.19; 5.93] | 2.11 [0.33; 13.54] | . |
| 0.26 [0.05; 1.44] | 0.42 [0.09; 2.07] | 0.18 [0.02; 1.46] | 0.53 [0.12; 2.37] | 0.03 [0.00; 1.04] | 0.37 [0.05; 2.87] | 0.43 [0.05; 3.75] | 0.47 [0.08; 2.88] | Corticoids+Topical Silicone Gel | . | . | . | . | . | 2.33 [0.39; 13.86] | . | . |
| 1.04 [0.12; 9.24] | 1.67 [0.27; 10.35] | 0.71 [0.06; 9.10] | 2.10 [0.26; 16.84] | 0.12 [0.00; 5.50] | 1.47 [0.12; 18.67] | 1.69 [0.12; 22.98] | 1.86 [0.18; 19.43] | 3.97 [0.35; 44.89] | Corticoids+Verapamil | . | . | . | . | . | . | . |
| 1.23 [0.11; 13.86] | 1.98 [0.17; 23.26] | 0.85 [0.06; 12.45] | 2.50 [0.26; 23.73] | 0.14 [0.00; 7.19] | 1.75 [0.12; 26.33] | 2.01 [0.13; 31.35] | 2.21 [0.18; 27.47] | 4.72 [0.32; 70.54] | 1.19 [0.06; 25.51] | Enalapril | . | . | . | . | . | . |
| 0.56 [0.08; 3.72] | 0.90 [0.13; 6.31] | 0.38 [0.05; 3.11] | 1.13 [0.21; 6.04] | 0.07 [0.00; 2.32] | 0.79 [0.08; 7.57] | 0.91 [0.09; 9.08] | 1.00 [0.13; 7.57] | 2.14 [0.23; 20.25] | 0.54 [0.04; 7.80] | 0.45 [0.03; 7.49] | Insulin | . | . | . | . | . |
| 0.76 [0.14; 4.15] | 1.22 [0.21; 7.09] | 0.52 [0.07; 4.11] | 1.54 [0.36; 6.55] | 0.09 [0.00; 2.97] | 1.08 [0.13; 8.75] | 1.24 [0.26; 5.99] | 1.36 [0.22; 8.57] | 2.91 [0.36; 23.38] | 0.73 [0.06; 9.26] | 0.62 [0.04; 8.95] | 1.36 [0.15; 12.40] | Pentoxifylline | . | . | . | 0.14 [0.01; 3.84] |
| 1.43 [0.31; 6.65] | 2.30 [0.41; 12.99] | 0.98 [0.12; 7.81] | 2.90 [0.67; 12.52] | 0.17 [0.00; 5.63] | 2.02 [0.26; 15.76] | 2.33 [0.27; 20.00] | 2.56 [0.42; 15.66] | 5.47 [0.69; 43.48] | 1.38 [0.11; 17.09] | 1.16 [0.08; 16.98] | 2.55 [0.28; 23.57] | 1.88 [0.24; 14.69] | Platelet Rich Plasma | . | 0.57 [0.11; 3.08] | . |
| 0.60 [0.15; 2.40] | 0.96 [0.24; 3.76] | 0.41 [0.06; 2.61] | 1.21 [0.39; 3.74] | 0.07 [0.00; 2.07] | 0.84 [0.17; 4.29] | 0.97 [0.14; 6.74] | 1.07 [0.26; 4.31] | 2.28 [0.46; 11.31] | 0.57 [0.06; 5.63] | 0.48 [0.04; 5.99] | 1.06 [0.14; 8.01] | 0.78 [0.12; 4.90] | 0.42 [0.07; 2.53] | Radiofrequency | 1.99 [0.31; 12.80] | . |
| 1.48 [0.46; 4.80] | 2.38 [0.63; 9.03] | 1.02 [0.18; 5.84] | 3.00 [1.17; 7.73] | 0.17 [0.01; 4.86] | 2.10 [0.41; 10.63] | 2.42 [0.39; 15.16] | 2.66 [0.69; 10.20] | 5.67 [1.01; 31.93] | 1.43 [0.15; 13.71] | 1.20 [0.10; 13.80] | 2.65 [0.39; 18.09] | 1.95 [0.35; 10.96] | 1.04 [0.23; 4.77] | 2.49 [0.65; 9.51] | Verapamil | . |
| 0.46 [0.06; 3.89] | 0.75 [0.08; 6.57] | 0.32 [0.03; 3.61] | 0.94 [0.14; 6.50] | 0.05 [0.00; 2.27] | 0.66 [0.06; 7.66] | 0.76 [0.07; 8.48] | 0.83 [0.09; 7.82] | 1.78 [0.15; 20.50] | 0.45 [0.03; 7.66] | 0.38 [0.02; 7.31] | 0.83 [0.06; 10.68] | 0.61 [0.06; 5.81] | 0.32 [0.03; 3.67] | 0.78 [0.08; 7.32] | 0.31 [0.04; 2.69] | Vitamin D |
